# Clinical and immunological spectrum of MHC class I deficiency: insights from a long-term cohort with two novel mutations

**DOI:** 10.3389/fimmu.2025.1675097

**Published:** 2025-10-07

**Authors:** Sule Haskologlu, Aydan Ikinciogullari, Candan Islamoglu, Sevgi Kostel Bal, Deniz Bayrakoglu, Serife Erdem, Zeynep Ceren Karahan, Omur Ardeniz, Caner Aytekin, Aylin Heper, Serdar Ceylaner, Figen Dogu

**Affiliations:** ^1^ Faculty of Medicine, Department of Pediatric Immunology and Allergy, and Hematopoietic Stem Cell Transplantation Unit, Ankara University, Ankara, Türkiye; ^2^ Department of Pediatric Immunology and Allergy, Gülhane Training and Research Hospital, Ankara, Türkiye; ^3^ Betul-Ziya Eren Genome and Stem Cell Center, Erciyes University, Kayseri, Türkiye; ^4^ Faculty of Medicine, Department of Medical Microbiology, Ankara University, Ankara, Türkiye; ^5^ Faculty of Medicine, Department of Internal Medicine, Division of Allergy and Immunology, Ege University, Izmir, Türkiye; ^6^ Department of Pediatric Allergy and Immunology, Dr. Sami Ulus Children's Health and Diseases Training and Research Hospital, Ankara, Türkiye; ^7^ Faculty of Medicine, Department of Medical Pathology, Ankara University, Ankara, Türkiye; ^8^ Intergen Genetics and Rare Diseases Diagnosis Research & Application Center, Ankara, Türkiye

**Keywords:** MHC class I deficiency, granulomatous skin lesions, uveitis, anti-rubella IgM positivity, follow-up

## Abstract

**Background:**

Major histocompatibility complex (MHC) Class I deficiency is a rare form of primary immunodeficiency that typically presents with recurrent sinopulmonary infections, bronchiectasis, and granulomatous skin lesions during late childhood or adolescence.

**Methods:**

This retrospective study describes the clinical, immunological, and long-term follow-up data of 11 patients diagnosed MHC Class I deficiency.

**Results:**

The cohort included 11 patients (6 males, 5 females) with a median age of 26 years (range 19–44). The median age at diagnosis was 19 years, with a diagnostic delay of 14 years. Bronchiectasis was seen in 10 patients, granulomatous skin lesions in 6, uveitis in 5, and nasal septum perforation in 3. All but one patient survived during a median follow-up of 11 years. HLA-ABC expression ranged from 0% to 73%, with persistently low mean fluorescence intensity (0.4–3.8). IgM levels were reduced in 7 patients. Ten patients were persistently positive for anti-rubella IgM, including all six with granulomatous skin lesions. Immunophenotyping revealed reduced CD3^+^ (n=2), CD4^+^ (n=3), CD8^+^ (n=3), CD19^+^ (n=5), CD3^−^CD16^+^CD56^+^ (n=3), CD19+ IgM-27+ IgD- (switched memory B cells) (n=7), and CD19+ IgM-27+ IgD+ (marginal zone B cells) (n=8). All patients had elevated γδ+ T cells, and NK cells were reduced in three. Seven patients had TAP1 and four had TAP2 mutations, with no significant genotype–phenotype differences.

**Conclusion:**

MHC Class I deficiency presents a broad clinical spectrum from asymptomatic to life-threatening disease. Granulomatous tissue damage and uveitis contributed to morbidity. Persistent rubella-specific IgM in most patients, including those without granulomas, is a novel serologic finding that may reflect altered antiviral immunity. Its clinical significance remains uncertain and, further studies with tissue-based viral detection are needed to clarify this observation.

## Introduction

Major histocompatibility complex (MHC) class I deficiency is a rare autosomal recessive form of inborn error of immunity (IEI) characterized by a reduced or absent expression of HLA class I molecules on the cell surface (1–5). MHC class I molecules are expressed by all nucleated cells, have roles in intracellular peptide antigen processing and presentation, thymocyte development in thymus, regulation of the activities of natural killer (NK) and γδ+ T cells. This deficiency is caused by genetic defects in the transporter associated with antigen processing (TAP) proteins; *TAP1*, *TAP2*, TAP-binding protein (TAPBP or tapasin), or β2-microglobulin (*B2M*) genes (4–9). TAP1 and TAP2 play an essential role in the antigen presentation pathway by translocating cytosolic peptides derived from proteasomal degradation into the endoplasmic reticulum lumen. In this location, the peptides are loaded into MHC class I molecules, which results in their presentation at the cell surface and recognition by CD8+ T cells or natural killer (NK) cells. TAPBP stabilizes TAP and associates with the MHC-I heavy chain. B2M is a component of the MHC-I molecule (9–11). MHC-I plays a crucial role in the positive selection of CD8+ T lymphocytes and the regulation of NK-cell homeostasis by supporting their development and subsequently suppressing NK-cell effector functions (11–13). In MHC Class I deficiency, HLA-ABC expression level typically ranges from 1% to 15% of normal. Although total CD8^+^ T-cell counts may be preserved, naïve CD8^+^ TCRαβ^+^ cells are markedly reduced, and expansions of γδ T cells, mucosa-associated invariant T (MAIT) cells, and invariant NKT cells have been reported (10–16). NK-cell numbers are often normal or increased, yet exhibit impaired cytotoxic function (11–14).

To date, nearly 30 patients with TAP1 and/or TAP2 deficiency have been reported, while tapasin deficiency has been described in one patient and or β2-microglobulin deficiency in two patients (6, 14–18). Patients with MHC Class I deficiency may be asymptomatic during infancy. Clinical manifestations typically emerge in the first decade, characterized by recurrent bacterial respiratory infections that can lead to chronic inflammatory lung disease and bronchiectasis (10, 17). Approximately half of patients develop skin lesions, which misdiagnosed as Wegener’s granulomatosis due to the absence of identifiable infectious agents and vasculitis-like features (15, 16, 18). Later studies suggested that these skin lesions result from immune dysregulation involving the accumulation of NK and γδ+ T cells (19). In recent years, persistent rubella vaccine strain has been detected in granulomatous skin lesions of patients with DNA repair defects and other inborn errors of immunity, including TAP1 and TAP2 deficiencies (20–24). These findings suggest that rubella virus may contribute to granuloma formation in the context of impaired immune regulation.

This study presents the largest long-term cohort of genetically confirmed TAP1 and TAP2 mutations to date, comprising 11 patients, and expands the clinical and immunological spectrum of MHC Class I deficiency by reporting two novel mutations, as well as novel findings such as reduced serum IgM levels, altered B-cell subsets, and prolonged rubella-specific IgM positivity - observed also in patients without granulomatous skin lesions, highlighting the need for increased awareness and further investigation.

## Materials and methods

### Patient data

We studied 11 patients (6 males and 5 females) diagnosed with MHC Class I deficiency from six different families, written informed consent was obtained from the patients and families according to the Declaration of Helsinki. The clinical, immunological and follow-up characteristics of the patients were evaluated retrospectively. Demographic and clinical data were collected, including age, gender, presenting symptoms, age at onset and diagnosis, family history, infectious agents, and findings from physical exams, pulmonary function test (PFTs), imaging, and pathology (when available). Laboratory evaluations included complete blood counts, immunoglobulin levels (IgG, IgA, IgM, total IgE), vaccine responses (anti-HBs, anti-Rubella IgG/IgM), ANA, and detailed lymphocyte subset analyses. Immunoglobulin levels and lymphocyte subset analysis results were evaluated according to the reference ranges of our laboratory (25, 26). Type I Interferon (IFN) measurement was performed in three patients. The follow-up of the patients, the treatments they received, and the findings at the last controls were evaluated.

### Genetic analysis

In the first four patients (P1–P4), genetic diagnosis was established by Sanger sequencing. In addition, complementation assays using EBV-transformed B cells were performed to confirm the functional impact of the mutations. The first two patients (P1 and P2) were previously reported, and a homozygous c.1312C>T (p.Arg438*) mutation in TAP1 was identified by cDNA and genomic sequencing (27). In the remaining seven patients, genomic DNA was extracted from peripheral blood collected in EDTA tubes. Based on clinical findings and markedly reduced HLA-ABC expression detected by flow cytometry, a targeted genetic analysis for MHC Class I deficiency was performed. All coding exons and exon-intron boundaries of the TAP1, TAP2, TAPBP, and B2M genes were analyzed using next-generation sequencing on the Illumina MiSeq platform. Sequence reads were aligned to the human reference genome (GRCh38/hg38), and variant calling was performed using MiSeq Reporter. Variants were annotated, filtered based on population frequency and predicted pathogenicity (e.g., SIFT, PolyPhen-2, MutationTaster), and classified according to ACMG guidelines. Following the diagnosis of each proband, cascade genetic testing and flow cytometric analysis were performed in Families I–III, including parents and siblings, whereas other families were evaluated only by flow cytometric analysis of HLA-ABC expression.

### Flow cytometric analysis

#### Peripheral blood lymphocyte subsets

Flow cytometric analysis was conducted using a Navios EX FlowCytometer (Beckman Coulter Corp. Miami FL, USA). T-cell subsets were defined as follows: total T cells (CD3^+^), CD4^+^ helper T cells (CD3^+^CD4^+^), CD8^+^ cytotoxic T cells (CD3^+^CD8^+^), naïve T cells (CD45RA^+^CCR7^+^), central memory T cells (CD45RA^−^CCR7^+^), effector memory T cells (CD45RA^−^CCR7^−^), and terminally differentiated effector memory T cells (TEMRA; CD45RA^+^CCR7^−^). Additionally, γδ T cells (CD3^+^TCRγδ^+^), NK cells (CD3^−^CD56^+^), the expression of HLA-ABC and HLA-DR and mean fluorescence intensities (MFI) of HLA-ABC were analyzed. B-cell subsets included total B cells (CD20^+^), switched memory B cells (CD19^+^IgM^−^CD27^+^IgD^−^), marginal zone B cells (CD19^+^IgM^+^CD27^+^IgD^+^), naïve B cells (CD19^+^IgM^+^CD27^−^IgD^+^), and activated B cells (CD19^+^CD38lowCD21low).

The methods for the lymphocyte activation test and the respiratory burst test can be found in the Supplementary material and methods.

### Serology tests

Rubella IgG and IgM levels were measured using the Elecsys Rubella IgG and Rubella IgM assays (Roche Diagnostics, Mannheim, Germany) on the cobas e 801 analyzers. The test method is described in the Supplementary Materials and Methods section.

### Type I ınterferon assay

Peripheral blood mononuclear cells (PBMCs) were isolated by density gradient centrifugation using Ficoll-Paque™. Cells (1 × 10^6^) were cultured in RPMI-1640 medium supplemented with 10% FBS and 1% penicillin–streptomycin, and incubated for 24 hours under two conditions: unstimulated and stimulated with 20 µg/mL Polyinosinic–polycytidylic acid [Poly I: C]. Total RNA was extracted using PureZOL™ reagent. RNA concentration and purity were assessed spectrophotometrically. cDNA was synthesized from 1 µg of RNA, and qRT-PCR was performed using SYBR^®^ Green Supermix. 18S rRNA served as the reference gene, and expression was analyzed via the 2^–ΔΔCt method. Details of the method are provided in the Supplementary Material and Methods.

## Results

### Patient characteristics

We evaluated 11 patients from six unrelated families (Family I: P1, P2; Family II: P3, P4; Family III: P5–P7; Family IV: P8, P9; Family V: P10; Family VI: P11). Two patients (P1 and P2) were previously reported (27). Mean age at last review was 30 years (range 19–44). Symptoms began at a mean age of 5 years (range 0.5–17), whereas mean age at diagnosis was 19 years (range 10–37), yielding a median diagnostic delay of 14 years (range 1–32). Ten of 11 patients were born to consanguineous parents; the remaining patient’s parents were non-consanguineous but came from neighboring villages. Before confirmation of MHC class I deficiency, five patients were followed up as asthma or chronic bronchitis, and two as cutaneous tuberculosis. Two patients’ selective IgA deficiency was resolved over the follow-up period. Baseline clinical data summarized in **Table 1**. Pedigrees, and variants are summarized in **Supplementary Figures S1A–F**.

**Table 1:** Clinical features and genetic analysis results of patients with MHC Class I deficiency.

	P1	P2	P3	P4	P5	P6	P7	P8	P9	P10	P11
Gender / Age	M / 36	F / 41	M / 21	F / 34	M / 19	M / 22	F / 26	F / 40	M / 44	M / 19	F / 23
Initial symptoms	Toxoplasmosis-associated retinitis and pneumonia, RTIs, skin wounds,	RTIs	RTIs, skin wounds,	RTIs	RTIs	RTIs	RTIs	RTIs, skin wounds,	RTIs	RTIs, skin wounds	RTIs, skin wounds
Symptom onset age (year)	0,5	10	5	17	0,5	1	1	1	10	1	11
Diagnosis age (year)	15	20	10	18	12	15	20	33	44	14	23
Consanguinity	Yes	Yes	Yes	Yes	Yes	Yes	Yes	Yes	Yes	Yes	Near villages
Genetic variants (hg38)	TAP1:c.1132C>T p.Arg378* NM_000593.6 chr6-32850436 G>A	TAP1:c.1132C>T p.Arg378* NM_000593.6 chr6-32850436 G>A	TAP2: NM_001290043.2 | ENST00000374897.4 Exon 6 |c.1023dup p.Val342Argfs*10 chr6:32832746 C>CG	TAP2: NM_001290043.2 | ENST00000374897.4 Exon 6 |c.1023dup |p.Val342Argfs*10 chr6:32832746 C>CG	TAP1:c.1132C>T p.Arg378* NM_000593.6 chr6-32850436 G>A	TAP1:c.1132C>T p.Arg378* NM_000593.6 chr6-32850436 G>A	TAP1:c.1132C>T p.Arg378* NM_000593.6 chr6-32850436 G>A	TAP2:c.1569del p.Gly525Aspfs*36 NM_001290043.2 chr6-32830332 CT>C	TAP2:c.1569del p.Gly525Aspfs*36 NM_001290043.2 chr6-32830332 CT>C	TAP1:c.1132C>T chr6-32850436 G>A p.Arg378* NM_000593.6	TAP1:c.781del chr6- p.Gln261Argfs*28 NM_000593.6 chr6:32852171 TG>T
ACMG classification	Pathogenic	Pathogenic	Likely pathogenic	Likely pathogenic	Pathogenic	Pathogenic	Pathogenic	Likely pathogenic	Likely pathogenic	Pathogenic	Likely pathogenic
Hearing loss	No	Yes	Yes	Yes	Yes	Yes	Yes	Yes	No	Yes	No
Granulomatous skin lesions / age at onset	Yes / 13	Yes / 32	Yes / 9	No	No	No	No	Yes / 32	No	Yes / 12	Yes / 11
Ocular findings	Uveitis, retinitis	Uveitis	Uveitis, conjunctival cyst	Uveitis, glaucoma	No	No	No	Uveitis, glaucoma	No	No	No
Nasal septum perforation	No	Yes	Yes	No	No	No	No	Yes	No	No	No
Treatment	IVIG, TMP-SMX	IVIG, TMP-SMX Posokanazol	IVIG, TMP-SMX, MSC, allo skin graft and flap surgeries	IVIG, TMP-SMX	IVIG, TMP-SMX, vorikonazol	IVIG, TMP-SMX	IVIG, TMP-SMX	IVIG, TMP-SMX	IVIG, TMP-SMX	IVIG, TMP-SMX	IVIG, TMP-SMX

### Genetic analyses

Given the consistent clinical phenotype and markedly reduced HLA-ABC surface expression and MFI values in all patients only targeted gene analyses were conducted. The first four patients (P1–P4) were analyzed by Sanger sequencing, while the remaining seven patients underwent targeted next-generation sequencing (NGS) including *TAP1*, *TAP2*, *TAPBP*, and *B2M*. A homozygous nonsense variant in *TAP1* (NM_000593.5:c.1312C>T; p.Arg438Ter), previously reported (26), was identified in P1 and P2 (FI), and also in unrelated patients P5, P6, and P7 (FIII), and P9 (FV). This pathogenic variant, located in exon 5, introduces a premature termination codon, likely resulting in nonsense-mediated mRNA decay (NMD) or a truncated, non-functional TAP1 protein. In P3 and P4 (FII), a single-nucleotide insertion (NM_000544.5:c.1022dupC) in TAP2 led to a frameshift (p.Ala341fs), disrupting the protein between transmembrane domains 7 and 8. In P8 and P9, a novel homozygous single-nucleotide deletion in *TAP2* (NM_000544.5:c.1569del; p.Gly524ValfsTer14) was identified, predicted to result in either NMD or a truncated, dysfunctional protein. In P11, a novel homozygous frameshift deletion in *TAP1* (NM_000593.5:c.781del; p.Gln261ArgfsTer28) was detected. All identified variants are consistent with autosomal recessive MHC class I deficiency and correlate with the immunologic phenotype. One heterozygous carrier identified through genetic testing manifested mild autoimmune features, whereas all other carriers and flow cytometry–screened relatives remained asymptomatic.

### Infections

All patients had recurrent sinopulmonary infections and otitis media. Bacterial pathogens most frequently isolated from sputum or BAL were *Streptococcus pneumoniae* (n = 3), *Haemophilus influenzae* (n = 2) and *Pseudomonas aeruginosa* (n = 2). Additional isolates included *Rothia mucilaginosa*, *Acinetobacter baumannii* and *Enterococcus* spp. Two patients (P2, P5) had *Aspergillus fumigatus* pneumonia documented by BAL culture; fungal pneumonia has not been previously reported in MHC-I deficiency. Viral infections comprised rhinovirus (P1), metapneumovirus (P2), and CMV viraemia with adenovirus pneumonia (P5). Childhood measles was reported anecdotally in P8 and P9 without laboratory confirmation. Prior to the initiation of IgRT, P9’s measles IgG value was found to be positive, while IgM was negative. CMV and EBV PCR loads remained below clinically significant thresholds during follow-up. P1 additionally developed *Toxoplasma gondii*–associated retinitis and pneumonia.

### Organ damage

#### Pulmonary manifestations

Bronchiectasis was present in 10/11 patients. Pulmonary‐function tests showed restrictive (n = 4), obstructive (n = 3) or mixed (n = 1) patterns; two patients (P1, P7) had severe restriction. Bronchiectasis and mucus plugging is shown in the chest CT scan of P7 (**Supplementary Figure S2**).

#### Granulomatous and vasculitic manifestations

**Figure 1:**
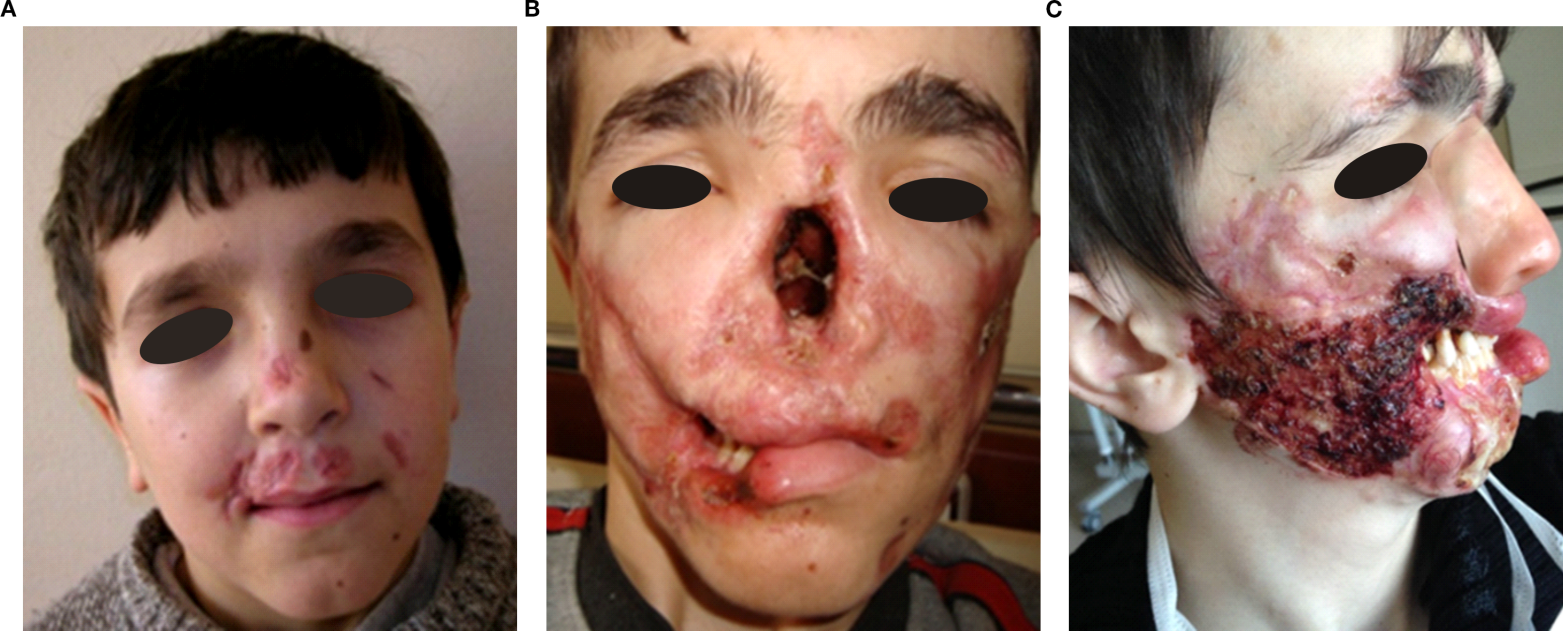
Chronological progression of granulomatous lesions in P3 from age 9 to 17. **(A)** Age 9: Initial facial skin lesions. **(B)** Nasal bone destruction and progression of lesions at age 12. **(C)** Progressive nasal bone and palate resorption, upper lip tissue loss, worsening skin lesions, and prosthetic nose implantation at age 17. The appearance of the skin wounds during active infection.

**Figure 2:**
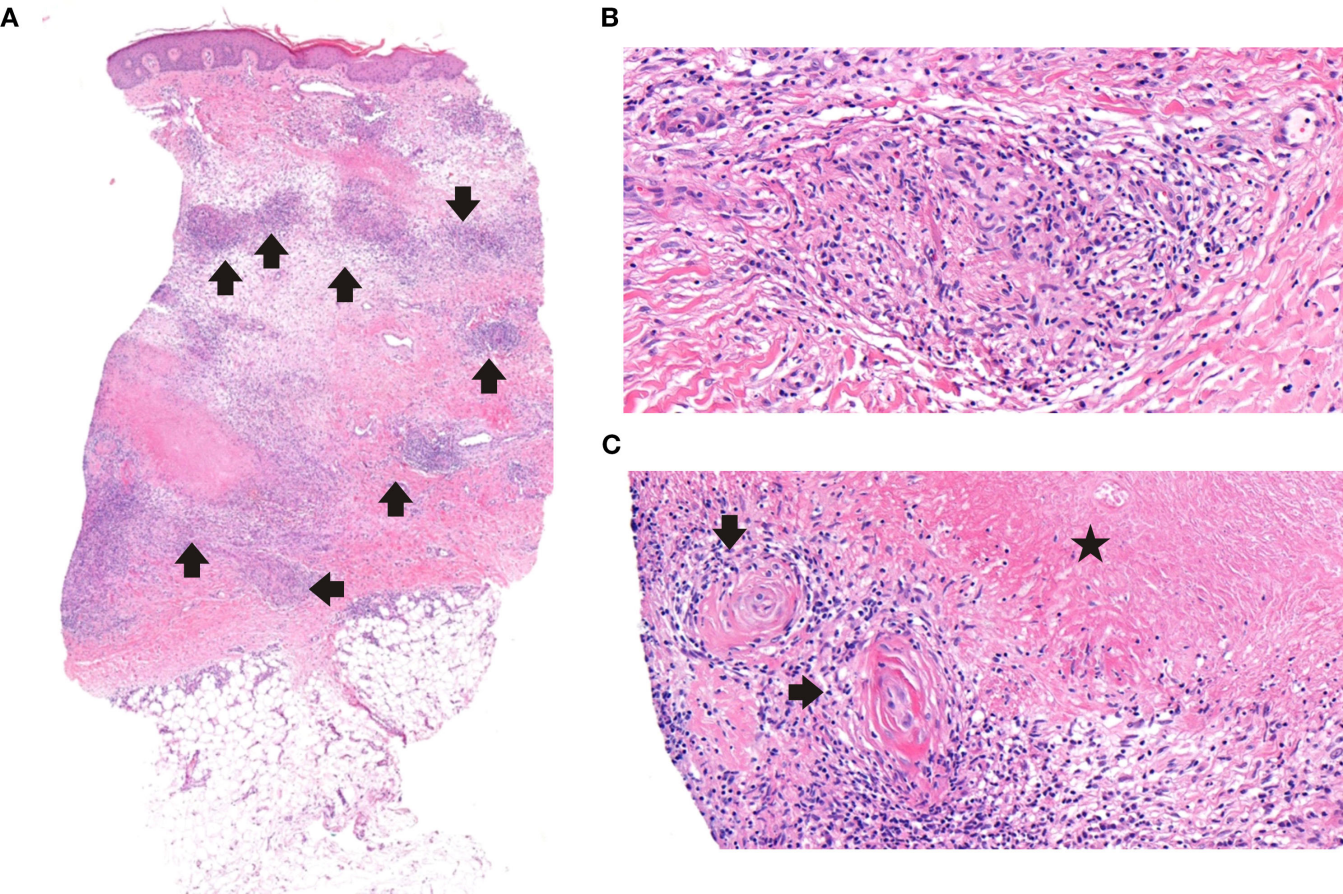
Histopathological features of cutaneous granulomas. **(A)** Granulomatous inflammation throughout the dermis with peripheral edema; mild extension into the superficial subcutaneous adipose tissue (H&Ex3.6). **(B)** Small non-necrotizing granulomas composed of histiocytes, few lymphocytes, and scattered eosinophils (H&Ex30.1)**. (C)** Small vessels adjacent to necrobiosis, display mild vasculitic changes with concentric wall thickening and sparse inflammatory cells (H&Ex25.9).

Granulomatous skin lesions occurred in six patients (54.5 %) (**Supplementary Figures S3-6**). Lesions were well-demarcated squamo-papular plaques, particularly on face and limbs. P3 developed refractory ulcers and facial tissue loss despite antibiotics, corticosteroids, surgery, (**Figure 1**). He also did not response to mesenchymal stem cell therapy, either (**Supplementary Figures S7**, **S8**). He died at the age of 21 due to sepsis, which was secondary to skin infections. All skin lesions were confirmed histologically via biopsy. No bacteria, mycobacteria or fungal agents were found in the biopsies. Histopathological examination revealed granulomatous inflammation and vasculitic changes in one patient, whose representative skin biopsies are shown in **Figure 2**. P8 showed lower-limb skin discoloration and oedema consistent with vasculitis (**Supplementary Figure S9**).

#### Ear-nose-throat involvement

Eight patients developed mastoiditis due to recurrent otitis media, resulting in conductive hearing loss that required tympanostomy tube placement. All patients exhibited rhinolalia.

Nasal septum perforation occurred in three patients (P2, P3, and P8). While nasal skin lesions were present in P2 and P3, they were absent in P8. In P3, progressive tissue destruction led to autoamputation of the nasal bone, cartilage, soft tissue, and most of the lips, severely affecting feeding and facial integrity. (**Figure 1**).

#### Ocular disease

Five patients developed ocular complications. Visual loss was irreversible in all five. Two (P4 and P8) required glaucoma surgery, and P3 underwent excision of a necrotizing granulomatous lesion. Three patients displayed heterochromia and enophthalmos (**Supplementary Figure S10**).

### Immunological findings

#### Cellular immune system assessment

Total lymphocyte counts were within normal range in all but two patients (P3 and P4). HLA−ABC surface expression ranged from 0% to 73% across patients. Despite some individuals exhibiting relatively higher expression percentages, mean fluorescence intensities (MFI) remained consistently 7- to 30-fold lower than in healthy controls (patients: 0.6–3.8 *vs*. controls: 25–40) throughout follow-up (**Table 2**, **Supplementary Figure S11**).

**Table 2:** Detailed immunophenotyping of patients with MHC class I deficiency at diagnosis and follow-up*.

	P1	P2	P3	P4	P5	P6	P7	P8	P9	P10	P11	Age references
WBC (mm^3^)	7900 / 7330	5390 / 5530	4000 / 4300	5240/ 7500	11400 / 5980	8620 / 9750	13400/ 6860	10900 / 4030	7680 / 7500	9800 / 8250	7530/ 5220	4500-11000
TLC (mm^3^)	1700 / 1930	1870/ 1770	900 / 1200	1300 / 1190	3260 / 2280	3300 / 2310	2290 / 1620	1930 / 1790	2970 / 3200	4620 / 2580	1500 / 1800	1500-4000
TNC (mm^3^)	2200 / 4980	2940 / 3230	3800 / 2300	3740 / 5500	6690 / 2770	4870 / 6240	10100 / 4410	8030 / 1650	3850 / 3400	4120 / 4820	3170 / 2900	1800-7700
Hb (g/dl)	12,7/ 14	11,7	12,6 /10	13 / 13	12,2 /14	15,2/ 16	11,4/ 12	10,3/ 11	15,7/ 15	13,1/ 14	12,1/12	12-15,5
Thrombocyte (mm^3^)	359000 / 362000	372000 / 365000	258000 / 242000	266000 / 338000	243000 / 259000	269000 / 201000	413000 / 194000	427000 / 195000	280.000 / 276000	636.000 / 377000	358.000 / 269000	150000-450000
IgG (mg/dl)	1460 / 1110	2400 / 2860	1690 / 2220	1770 / 1870	2560 / 2080	2110 / 1920	1820 / 2080	942 / 2400	1900 / 1617	1250 / 2230	1340/ 1500	876-2197
IgA (mg/dl)	255 / 189	599 / 610	250 / 396	312 / 389	419 / 398	<6 / 97	326 / 296	64 / 131	242 / 228	147 / 173	395 / 404	96-465
IgM (mg/dl)	49 / 38	59 / 58	31 / <17	82 / 74	94 / 86	36 / 44	178 / 201	130 / 110	100 / 85	41 / 27	47 / 55	88-322
T. IgE (IU/mL)	5,4 / 42	10	82	1,6	nd / 29	7/ nd	7	1,6	nd	8	25	2-214
Isohemag. titre	AB Rh+	AB Rh+	¼+	1/32+	1/32+	1/64+	AB Rh+	1/64+	1/64+	1/8+	1/8+	
ANA	No	No	No	nd	+1	No	Spotted +1	Spotted +1	nd	+1	+1	
CD3+16-56- (%, mm^3^)	69 (1173) / 73 (1408)	66 (1234) / 49 (867)	50 / (450) / 64 (768)	66 (858) / 70 (833)	53 (1727) / 58 (1320)	58 (1914) / 51 (1178)	74 (1694)/ 66 (1069)	70 (1351) / 54 (966)	68 (2019) / 65 (4875)	64 (4118) / 53 (1360)	88 (1320) / 76 (1368)	(58-82) / (1100-4100)
CD3+CD4+ (%, mm^3^)	35 (595) / 45 (868)	32 (598) / 31 (548)	54 / (486) / 46 (552)	48 (624) / 57 (678)	37 (1206) 36 (820)	39 (1287) / 38 (877)	46 (1053) / 36 (583)	41 (791) / 20 (358)	26 (772) / 28 (2100)	44 (2032) / 36 (1026)	55 (825) / 48 (864)	(26-48) / (600-2400)
CD3+CD8+ (%, mm^3^)	31 (527) / 19 (366)	36 (673) / 25 (442)	9 (81) / 6 (72)	16 (208) / 13 (155)	10 (326) / 16 (364)	15 (495) / 14 (323)	32 (733) / 32 (518)	67 (1293) / 52 (930)	41 (1205) / 37 (2775)	14 (647) / 15 (427)	25 (375) / 31 (558)	(16-32) / (400-1500)
CD3-16+56+ (%, mm^3^)	12 (204) / 13 (250)	13 (243) / 27 (477)	10 (90) / 15 (180)	18 (234) / 15 (178)	7 (228) 8 (159)	18 (594) / 28 (646)	9 (206) / 12 (194)	3 (58) / 6 (107)	11 (326) / 13 (416)	28 (1633) / 22 (627)	3 (45) / 9 (162)	(8-30) / (200-1000)
CD3-16+56+ ^bright^	3,6	1,6	1,9	1,3	0,7	1,5	0,8	1,5	2	5	0,7	
CD3-16+56+ ^dim^	9,6	21	9	17	8	18,9	13,7	32	16,7	21,4	15,8	
CD19+ (%, mm^3^)	9 (153) / 10 (193)	9 (168) / 12 (212)	33 (297) / 25 (300)	11 (143) / 9 (107)	32 (1043) / 28 (638)	14 (462) / 12 (277)	10 (229) / 15 (243)	14 (270) / 12 (214)	6 (178) / 5 (375)	10 (462) / 7 (199)	8 (120) / 6 (108)	10-30 / (200-1400)
HLA -ABC (range, %)	8-25	2-56	0,6-10	6-12	13-73	22-52	10-13	6-9	42-31	0	5-18	100
HLA-ABC MFI	1,24	0,44	0,6	1,22	2	3,8	3,8	2,9	3,7	2,1	2,5	25-30
HLA DR (%)	27 / 22	18 / 21	37 / 33	18 / 22	37	26 / 16	36	20 / 15	16 / 11	15 / 9	11 / 11	(16-35)
TCR γδ (%)	16	18/ 12	24/ 20	5/ 5	17-16	13 / 10	15	8 / 11	13 / 15	8/ 10	17/19	<5
CD45RA+CD31+ (%)	42 / 29	18 / 16	22/ 20	6 /6	45-26	33 / 14	26 / 9	27 / 18	14 /10	34/ 31	27/ 23	7-100
CD4+/CD8+ ratio	1,12	0,88	6	3	3,7	2,6	1,43	0,61	0,63	3,1	2,2	1-3
CD4+CD45RA+CCR 7+ (Naive Th) (%)	33 /	24 / 26	nd	6,6 / 6	35 / 31	20 / 16	21 / 18	21 / 20	20 / 19	38,6 / 29	39	32,7 (20,9-49)
CD4+CD45RA- CCR 7+ (Centr. memory Th) (%)	30	33 / 63	nd	49,3 / 53	45-43	43 / 31	62 / 64	21 / 21,7	36 / 38	25,4 / 30	19	33 (20,8-45,6)
CD4+CD45RA- CCR 7- (Effect. memory Th) (%)	36	37 / 8	nd	42,6 / 38	19-25	36 / 52	16 / 16	54 / 54	40/36	34,5 /31	29	25,1 (13,9-33,6)
CD4+CD45RA+CCR 7 - (TEMRA Th) (%)	1,4	6 / 2	nd	1,4 / 1,8	0,6 / 0,6	1,2 / 1	0,9 / 2,5	2,7 / 3	3,4 / 5	1,5 / 2	12,6	4,9 (2-10,3)
CD8+CD45RA+CCR7+ (Naive Tc) (%)	18	16 / 9	nd	25,3 / 22	13-10	36 / 36	17 / 2,3	4,3 / 4	22 / 17	60 / 51	14	29 (13,9-43,7)
CD8+CD45RA- CCR7+ (Central memory Tc) (%)	25	17 / 29	nd	19,7 / 24	29-13	23 / 18	27 / 3	1,4 / 1,2	30 / 25	9,3 / 16	13	3,4 (1,5-7,4)
CD8+CD45RA- CCR7- (Effect. memory Tc) (%)	47	55 / 52	nd	36,7 / 38	45-52	20 / 26	43 / 72	29,4 / 30	33,7 / 37	12,1 / 15	62,7	43,8 (25-56,9)
CD8+CD45RA+CCR7- (TEMRA Tc) (%)	10	13 / 10	nd	18,2 / 17	12-25	21 / 20	14 / 23	64,9 / 62	13,2 / 12	18,4 / 19	10	23,3 (9-44,7)
CD19+ IgM+27- IgD+ (Naive B) (%)	91,6	78,8 / 73	nd	60,3 / 58	91 / 78	77 / 74	81 / 85	89 / 82	68,7/ 70	80,8 / 50	81	(62,2-76,2)
CD19+ IgM-27+ IgD- (switched memory B) (%)	2,4	3,8 / 4	nd	20,8 / 19	2 / 4	9 / 8	4 / 3	0,8/ 1	4,9 / 4,2	4,5 / 3,3	6,8	(6,5-29,2)
CD19+ IgM-27+ IgD+ (marginal zone B) (%)	2,7	2,5 / 2	nd	8,3 /9	2 / 2	7 / 7	4 / 3	3,4 /3	6,6 / 6	3,2 / 2,7	8,4	(7,2-30,8)
CD19+ CD38 Low CD21 Low (Active B) (%)	1,9	12,2 / 14	nd	25,2 / 16	1,7 / 4	6 / 4	10 / 9	23 / 16	5,7 / 8	3,8 / 6,7	4,4	(1,1-6,9)
Lymphocyte activation response to PHA and anti-CD3	Normal	Normal	Normal	Normal	Normal	Normal	Normal	Normal	Normal	Normal	Normal	
HLA genotype	Homozyg.	Homozyg.	Homozyg.	Homozyg.	Homozyg.	Homozyg.	Homozyg.	Homozyg.	Homozyg.	Homozyg.	nd	
Burst test	nd	nd	nd	Normal	Normal	Normal	Normal	nd	Nd	Normal	nd	

*: The initial value indicates the value at the time of diagnosis, while the second value indicates the value at the last follow-up visit.

nd: not determined

T−cell subsets showed reduced CD3^+^ cells in two patients and reduced CD8^+^ cells in four (2/4 TAP2−deficient; 2/7 TAP1−deficient). All patients had elevated γδ+ T cell percentages (8–24 %). NK cells were reduced in three patients, with a predominance of CD56^dim^ phenotype. Review of immunological parameters at the latest follow-up revealed no significant changes in CD3^+^ T lymphocyte counts. In one patient’s CD4^+^ T cell counts normalized while CD8^+^ T cells decreased, in another patient, CD8^+^ T cells slightly increased, reaching normal levels.

#### Humoral immune system assessment

IgG and IgA levels were normal, while IgM levels were reduced in seven patients. Assessment of pathogen-specific humoral responses during natural infections revealed detectable IgG and IgM levels in several patients. Patient 1 had a prior Toxoplasma infection, with positive anti-toxoplasma IgM and IgG at the time of infection, although both titers declined over time. Patients 8, 9, 10, and 11 demonstrated positive IgG against measles and mumps, with negative IgM, while patients 5, 9, and 11 had detectable anti-CMV IgG. Pre-IgRT responses to pneumococcal, tetanus, and diphtheria vaccines were not available. B−cell analysis revealed low total CD19^+^ counts in five patients, decreased switched−memory B cells in seven, reduced marginal−zone B cells in eight, and increased naïve B cells in eight. After diagnosis, all patients were initiated on IgRT. During follow-up, IgG levels remained within or above the normal range. IgA levels remained largely unchanged, and two additional patients showed slightly decreased IgM levels at the latest follow-up. No notable changes were observed in other T or B lymphocyte subsets. Detailed immunophenotypes are provided in **Table 2**.

#### Rubella serology

Wild−type rubella infection was not reported in any patient. Seven patients (P1, P2, P3, P4, P8, P9, P11) were unvaccinated for rubella, and four (P5, P6, P7, P10) had only a single dose of the vaccination. Prior to the initiation of IgRT, anti−rubella IgG was positive in seven tested patients—three unvaccinated adults (P4, P9, P11), four single−dose MMR recipients. In three patients who hadn’t received the rubella vaccine, we detected very high anti-rubella IgG levels (>10,000 IU/mL in P4 and P9, and 8,766 IU/mL in P11) before starting IgRT and all of these patients had a 100% avidity. All those who received one dose of the vaccination tested positive for anti-rubella IgG prior to IgRT. However, their IgG titers were not extremely high and were similar to those in the healthy population. Remarkably, anti−rubella IgM was detected in 10 of 11 patients. Seven unvaccinated adults (born before 2006; P1, P2, P3, P4, P8, P9, P11), persistent IgM positivity over a mean of three years, notably including all patients with granulomatous skin lesions. Rubella IgM positivity was also detected in four other patients without granulomas. All these serological and clinical findings suggest that there is a continuous antigenic stimulus in patients and a broadly dysregulated specific response to rubella virus. Furthermore, anti-IgM levels for measles and mumps were negative in all patients. Rubella serology results are summarized in **Table 3**.

**Table 3:** Rubella antibody results of patients with MHC class I deficiency.

Patient No	MMR vaccine status	Anti- Rubella IgG before IgRT (Positive: >10 IU/ML)	Anti-Rubella IgM (negative:<1 COI)	Anti-Rubella IgM Persistence period (years)	Granulomatous skin lesion	Uveitis
P1	Not vaccinated	nd	4	5	Yes	Yes
P2	Not vaccinated	nd	4,3-5,9	5	Yes	Yes
P3	Not vaccinated	nd	1,81	5	Yes	Yes
P4	Not vaccinated	>10000	1,6-4,3	1	No	Yes
P5	One dose	164	10,5	2	No	No
P6	One dose	359	<1	Negative	No	No
P7	One dose	> 500	10,9--22	5	No	No
P8	Not vaccinated	nd	10,7	6	Yes	Yes
P9	Not vaccinated	>10000	7,79	1	No	No
P10	One dose	>50	2,24	1	Yes	No
P11	Not vaccinated	8766	2,01	1	Yes	No

#### Type I interferon signaling

Baseline expression of IFN−α/β–stimulated genes (ISGs) was low in two patients without granulomas (P5, P7) but elevated in P8, who had rubella−associated granulomas. Poly−I:C stimulation increased IFN−response gene transcription in all three patients (**Supplementary Figures S12**-**14**).

***ANA levels*** measured for autoimmunity screening were +1 positive in five patients (P5, P7, P8, P10 and P11) but negative in all other patients.

#### Treatment and outcome

All patients received immunoglobulin replacement therapy (IgRT) and trimethoprim–sulfamethoxazole prophylaxis. Targeted antibiotics and surgical interventions were used as required. One patient (P3) died of sepsis related to progressive cutaneous ulcers; the remaining ten are alive after a median follow−up of 11 years (range 6 months–22 years). Most long−term morbidity stemmed from chronic lung disease, while progressive granulomatous skin lesions and uveitis also contributed substantially.

## Discussion

MHC class I deficiency is a rare autosomal recessive inborn error of immunity caused by mutations in genes involved in antigen processing and peptide loading (28). While its hallmark features include recurrent sinopulmonary infections and granulomatous inflammation, the clinical spectrum is broader and remains under-characterized due to the limited number of reported cases. In this study, we describe 11 genetically confirmed patients the largest cohort to date with detailed long-term clinical and immunological follow-up, highlighting key phenotypic and immunologic patterns that expand current understanding of this condition.

In our cohort, symptom onset occurred early (mean 5.2 years; six patients with lower RTIs before age 1), yet the mean age at diagnosis was 19 years, reflecting a delay of 14 years. This prolonged diagnostic latency likely contributes to irreversible pulmonary damage: by confirmation, 10/11 patients had developed bronchiectasis, despite the absence of profound hypogammaglobulinemia. Recurrent bacterial infections most commonly S. pneumoniae, P. aeruginosa and H. influenzae and chronic inflammation drive airway remodeling and progressive bronchiectasis, underscoring the need to consider MHC Class I deficiency in any child with early, recurrent RTIs (6, 7). Chronic sinusitis, mastoiditis requiring tympanostomy tubes, and nasal septum perforation were prominent ENT complications. Nasal septal perforation occurred in three patients, significantly impairing their quality of life. MHC class I deficiency should be considered in children with recurrent purulent ear-nose-throat infections (5, 19).

Although the fungal and viral infections observed in our cohort do not suggest a consistent predisposition, reporting such cases may help expand the clinical spectrum of MHC class I deficiency and guide future research, particularly regarding antiviral immunity. TAP1 is involved not only in MHC class I antigen presentation but also in antiviral defense by enhancing IFN-β production via the TBK1/IRF3 pathway and inhibiting viral entry (29). Nonetheless, severe viral infections are uncommon, possibly due to compensatory mechanisms such as γδ+ T cell expansion, NK cell function, ADCC, and MHC II–mediated immunity (6, 10, 11, 19)

Cascade genetic and flow cytometric testing of family members revealed that most carriers remained asymptomatic, with only one heterozygous individual exhibiting mild autoimmune features. All siblings identified via cascade testing were initially nearly asymptomatic but later developed characteristic manifestations. These observations underscore the spectrum of clinical presentation even among individuals carrying the same pathogenic mutation. The inclusion of HLA-ABC expression analysis in routine lymphocyte subset evaluation played a pivotal role in expanding this cohort, enabling the identification of five additional patients through targeted family screening following the index case.

Cutaneous granulomatous lesions can be the first clinical sign of MHC Class I deficiency (30–32) and are sometimes misdiagnosed as Wegener’s granulomatosis or cutaneous tuberculosis because of their vasculitic appearance (17, 18). A pivotal turning point came when Bodemer et al. identified the rubella RA27/3 vaccine strain in granulomas from three IEI patients by transcriptomics and RT−PCR, later confirming rubella capsid protein by immunofluorescence (20). A blinded follow−up study detected rubella antigen in granulomas from 19 additional IEI cases (21). Although the phenomenon was first recognized in DNA−repair disorders, rubella−associated granulomas have since been reported across diverse IEIs and may emerge weeks to decades after MMR vaccination (22, 23). Wild−type rubella granulomas have also been described in adults with common variable immunodeficiency and other mild immune defects, suggesting that rubella virus can persist for decades and manifest when immune control wanes (32–34).

Previous reports relied on direct viral detection (RT−PCR, transcriptomics, immunohistochemistry) (33, 34). A recently published study revealed that four patients with TAP deficiency found chronic cutaneous granulomatous lesions associated with RuV infection. All patients were IgM negative and strongly IgG positive for rubella and their IgG antibody levels were much higher than the general population’s. Wild-type RuV was detected in two patients, and a vaccine-derived strain was detected in one. Rubella PCR results of skin biopsies of another patient were negative. However, it was noted that there is a strong presumption of an association with rubella virus based on the extremely high IgG titer in the patient with TAP deficiency (35).

Our study adds a novel serologic observation: persistent anti−rubella IgM positivity in ten of eleven patients, including all six with cutaneous granulomas (four unvaccinated) and five of the six who developed uveitis. IgM persistence for a mean of three years and implies failure of viral clearance or ongoing antigenic stimulation. However, since our observations have not been confirmed by tissue evidence (e.g., PCR, immunohistochemistry), they are descriptive rather than definitive. Previous studies have also reported sustained rubella IgM responses in primary immunodeficiency patients following MMR vaccination, interpreted as a marker of impaired clearance of vaccine-derived virus (36). Prospective studies are needed to determine whether persistent IgM can serve as a surrogate marker of viral persistence and potentially predict granuloma formation.

Importantly, we observed significantly elevated anti-Rubella IgG levels in unvaccinated patients, whereas IgG levels in vaccinated individuals were comparable to those in the healthy population. This finding raises the possibility that the immunological response to wild-type rubella virus differs from that induced by the vaccine strain.

The latency between rubella virus exposure and granuloma development may range from weeks to decades, as shown in previous studies (37), complicating timely diagnosis. In our cohort, cutaneous granulomas appeared between ages 9 and 32.

Extracutaneous rubella virus (RuV)-associated granulomas have been reported in various organs, including the liver, brain, lungs, and bone marrow. In one study, RuV-infected neutrophils (RVC^+^) were identified in the bone marrow of most patients with IEI, suggesting that bone marrow may serve as a viral reservoir and contribute to systemic dissemination via myeloid cells (34).

In our cohort, five patients developed uveitis and progressive visual loss; notably, four of them also had granulomatous skin ulcers. Congenital rubella infection is well known to cause ocular abnormalities such as microphthalmia, cataracts, retinopathy, and glaucoma, with prolonged viral shedding during infancy. Additionally, persistent wild-type RuV infection in immunocompromised or elderly individuals has been associated with less frequent complications, including rubella encephalitis, Fuchs’ uveitis, and arthritis (38–41). All five patients with uveitis tested positive for anti-rubella IgM, and three of them were unvaccinated. Although direct detection of RuV in ocular or systemic tissues was not performed in our study, the coexistence of persistent IgM and clinical findings supports the possibility of rubella virus involvement. Future investigations incorporating PCR or immunohistochemistry on tissue biopsies are warranted to clarify the etiologic relationship and explore the potential for RuV to cause extracutaneous manifestations—including ocular involvement—in patients with MHC class I deficiency.

MHC class I expression can be partially restored by adding exogenous peptides with appropriate specificity. de la Salle et al. showed that vaccinia virus–mediated TAP1, but not TAP2, expression restores MHC-I surface levels in TAP1-deficient cells (4). Interestingly, Tsilifis et al. found that patients with large TAP1/2 deletions retained up to 23% of normal MHC-I expression, suggesting TAP-independent peptide loading or variable stability of peptide-free MHC-I molecules (42).

In our cohort, HLA-ABC expression levels, assessed by flow cytometry, varied widely among patients, ranging from undetectable (0%) to as high as 72%, nevertheless, MFI consistently remained markedly lower than in healthy controls throughout follow-up. These values did not correlate with genotype or clinical severity and were consistent across multiple time points in each patient. All patients fulfilled the immunophenotypic, genetic, and clinical criteria for MHC class I deficiency, regardless of their HLA-ABC surface expression level. This emphasizes that even patients with relatively higher HLA-I expression may present with severe and persistent immunodeficiency.

Lymphopenia is rare in patients with MHC class I deficiency. Although absolute lymphocyte counts are often normal, mild reductions in T-cell ratios have been reported. In some cases, CD4^+^ and CD8^+^ T cells may decline progressively, and CD8^+^ T-cell proportions can range widely—from 7–20% to as high as 88% of PBMCs—with notable fluctuations over time (6).

In a study by Darazam et al., two patients with TAP2 deficiency underwent detailed immunophenotyping. B-cell subsets were generally normal, except for a mild increase in plasma cells. NK cell counts particularly CD56^dim^ subsets, were elevated. CD4+ T cell subsets were within normal ranges, but CD8+ naïve T cells were reduced (14).

Despite the central role of CD8^+^ T cells in viral immunity, overt or persistent viral infections were infrequent in our cohort. One possible explanation is the relative preservation of total lymphocyte counts in most patients, which may contribute to partial immune compensation. Additionally, compensatory mechanisms such as γδ T-cell expansion, NK-cell function, and antibody-mediated clearance may help contain common viral pathogens, as proposed in previous studies (6, 10, 11, 30).

In our cohort, lymphopenia was observed only in two siblings. While IgG and IgA levels were normal, seven patients had reduced IgM levels—a rarely reported finding aside from Maeda et al. (1985) (43) Pre-IgRT humoral immunity appeared largely preserved, as several patients demonstrated pathogen- or vaccine-specific IgG/IgM prior to IgRT. These findings suggest that B cells can mount specific antibody responses despite MHC class I deficiency, although systematic assessment of vaccine responses was not performed. CD4^+^/CD8^+^ ratios varied, with normal, elevated, or decreased values across patients. Notably, CD8^+^ central memory T cells were increased in eight patients, while naive CD8^+^ T cells were reduced in two, and TEMRA helper T cells were decreased in five. B-cell profiling showed increased naïve B cells in eight patients, reduced switched memory B cells in seven, and decreased marginal zone B cells in eight. Altered T-cell subsets likely reflect impaired MHC class I–mediated selection and differentiation. In contrast, the observed decreased IgM levels and B-cell abnormalities—including reduced switched memory and marginal zone B cells—may indicate peripheral dysregulation of B-cell maturation or class-switching mechanisms. During follow-up, immunoglobulin levels under IgRT were consistently within or above the normal range for IgG, remained stable for IgA, and showed only minor deviations for IgM. T and B lymphocyte subsets remained largely stable, with only minor fluctuations in CD4^+^ and CD8^+^ T cell counts in individual patients.

Type-I IFN signaling varied according to rubella−associated pathology. In patients without granulomas (P5, P7), baseline expression of IFN−α, IFN−β1, and interferon−stimulated genes (IFI44L, CXCL9, IFIT1, RSAD2, IFI27) was lower than in controls, with significant upregulation upon Poly−I:C stimulation. Conversely, P8—who developed rubella−associated granulomas—showed elevated baseline ISG expression that further increased after stimulation. These data suggest that TAP defects may differentially impact antiviral IFN responses, potentially influencing rubella persistence and granuloma formation.

Our cohort revealed distinctive humoral alterations in MHC class I deficiency, including persistently low IgM and reduced switched memory and marginal zone B-cell subsets, despite largely preserved antiviral titers. Importantly, 10 of 11 patients exhibited persistent rubella-specific IgM, suggesting chronic antigenic stimulation in the context of impaired CD8^+^ T-cell immunity. In three patients evaluated before IgRT, markedly elevated rubella-specific IgG levels were observed despite no documented history of MMR vaccination or natural infection. Similar findings of high or normal antibody titers against common viral pathogens (measles, mumps, influenza, herpes, varicella, EBV, CMV) have also been reported in MHC class I deficiency (6).

Type I interferon responses may further shape these patterns. Experimental and clinical evidence indicates that rubella infection activates IFN signaling pathways essential for viral control (44). In our cohort, patients without granulomas showed reduced baseline type I IFN expression, although inducibility upon stimulation remained intact, whereas the single patient with granulomatous lesions exhibited persistently elevated ISG activity, suggestive of chronic type I IFN signaling. This pattern is compatible with sustained STAT1 activation—indirectly inferred from ISG upregulation—which has been shown in murine models and in patients with STAT1 gain-of-function mutations to impair switched memory and marginal zone B-cell development (45–48). Taken together, the coexistence of abnormal IFN responses, alterations in B-cell subsets, persistently positive rubella-specific IgM, and low serum IgM levels likely reflects a dysregulated but ongoing antiviral response in the context of inadequate T-cell–mediated clearance. This pattern may help explain the immune heterogeneity observed in MHC class I deficiency. Clinically, the presence of such immunologic alterations in patients who also develop granulomatous skin lesions or uveitis may serve as an early indicator of underlying inborn errors of immunity. In these cases, evaluation for HLA-ABC expression and TAP1/2 sequencing should be considered. Prospective studies are warranted to determine whether these features represent intrinsic consequences of MHC class I deficiency, predispose to persistent viral infections such as rubella, or both, and to guide targeted antiviral or anti-inflammatory strategies.

Although IgRT and trimethoprim-sulfamethoxazole prophylaxis reduced the frequency of RTIs, they did not prevent the progression of bronchiectasis, nasal perforation, or the development of granulomatous lesions.

Various treatment strategies for granulomatous skin lesions have been attempted, yet their efficacy remains limited. In our cohort, anti-tuberculosis therapies and systemic or topical corticosteroids were largely ineffective, while mesenchymal stem cell therapy and surgical interventions provided only transient improvement in a single patient. Similarly, in our cohort, nitazoxanide in a SCID-Artemis patient and anti-TNF therapy in an ataxia-telangiectasia patient provided only minimal or temporary benefit. Importantly, IgRT did not prevent lesion development or progression. These observations align with previous reports showing that antiviral agents such as nitazoxanide and ribavirin display *in vitro* activity but limited clinical efficacy, and those immunosuppressive approaches generally fail to resolve granulomatous disease (49–52).

Other modalities including chloroquine, psoralen plus ultraviolet A (PUVA), and thalidomide, have also been tried with inconsistent benefit, the latter being discouraged due to the risk of serious complications, especially in patients with bronchiectasis (53). Overall, the rarity of granulomatous lesions, absence of a curative treatment, and lack of a standardized therapeutic protocols represent major clinical challenges. Experience with HSCT is limited and variable, but currently represents the only potentially curative intervention (30, 42, 54).

While our cohort represents the largest series of genetically confirmed MHC class I deficiency to date, the high degree of familial clustering may limit the generalizability of our findings. Nevertheless, considerable variability in clinical and immunologic features was observed even among siblings carrying the same mutations, suggesting that additional modifiers. The clinical relevance of certain immunologic differences reported here will become clearer with larger and more diverse cohorts.

## Conclusion

MHC class I deficiency remains a rare and underdiagnosed IEI, frequently presenting with chronic RTIs, granulomatous skin lesions, and delayed diagnosis. In this large, long-term cohort, we identified hallmark findings such as markedly reduced HLA-ABC expression, decreased IgM levels, and alterations in T- and B-cell subsets. Although we could not confirm tissue-level rubella infection, the persistence of anti-rubella IgM—particularly in patients with granulomas and uveitis— together with significantly elevated IgG levels in unvaccinated patients compared to vaccinated ones may indicate an ongoing or dysregulated viral immune responses and warrants further investigation. Since MHC class I deficient patients may remain asymptomatic during infancy, establishing the diagnosis prior to MMR vaccination and preventing vaccine-related complications requires careful assessment of consanguinity and family history, along with appropriate immunological evaluation when indicated. Our results underscore the importance of early HLA-ABC screening, genetic testing, and family-based evaluation. Conventional therapies remain inadequate; while experience with HSCT is limited, it remains the only curative option and may guide future targeted interventions.

## Data Availability

The datasets presented in this study can be found in online repositories. The names of the repository/repositories and accession number(s) can be found in the article/**Supplementary Material**.

## References

[1] Touraine JL Betuel H Souillet G Jeune M. Combined immunodeficiency disease associated with absence of cell-surface HLA-A and -B antigens. J Pediatr. (1978) 93(1):47–51. doi: 10.1016/s0022-3476(78)80598-8, PMID: 650344

[2] Moins-Teisserenc HT Gadola SD Cella M Dunbar PR Exley A Blake N. Association of a syndrome resembling Wegener's granulomatosis with low surface expression of HLA class-I molecules. Lancet. (1999) 354(9190):1598–603. doi: 10.1016/s0140-6736(99)04206-3, PMID: 10560675

[3] de la Salle H Hanau D Fricker D Urlacher A Kelly A Salamero J. Homozygous human TAP peptide transporter mutation in HLA class I deficiency. Science. (1994) 65(5169):237–41. doi: 10.1126/science.7517574, PMID: 7517574

[4] de la Salle H Zimmer J Fricker D Angenieux C Cazenave JP Okubo M. HLA class I deficiencies due to mutations in subunit 1 of the peptide transporter TAP1. J Clin Invest. (1999) 103(5):R9–R13. doi: 10.1172/JCI5687, PMID: 10074495 PMC408129

[5] Gadola SD Moins-Teisserenc HT Trowsdale J Gross WL Cerundolo V. TAP deficiency syndrome. Clin Exp Immunol. (2000) 121(2):173–8. doi: 10.1046/j.1365-2249.2000.01264.x, PMID: 10931128 PMC1905688

[6] Zimmer J Andres E Donato L Hanau D Hentges F de la Salle H. Clinical and immunological aspects of HLA class I deficiency. QJM. (2005) 98(10):719–27. doi: 10.1093/qjmed/hci112, PMID: 16087697

[7] Donato L de la Salle H Hanau D Tongio MM Oswald M Vandevenne A. Association of HLA class I antigen deficiency related to a TAP2 gene mutation with familial bronchiectasis. J Pediatr. (1995) 127(6):895–900. doi: 10.1016/s0022-3476(95)70024-2, PMID: 8523185

[8] Yabe T Kawamura S Sato M Kashiwase K Tanaka H Ishikawa Y. A subject with a novel type I bare lymphocyte syndrome has tapasin deficiency due to deletion of 4 exons by Alu-mediated recombination. Blood. (2002) 100(4):1496–8. doi: 10.1182/blood-2001-12-0252, PMID: 12149238

[9] Ardeniz O Unger S Onay H Ammann S Keck C Cianga C. b2-Microglobulin deficiency causes a complex immunodeficiency of the innate and adaptive immune system. J Allergy Clin Immunol. (2015) 136(2):392–401. doi: 10.1016/j.jaci.2014.12.1937, PMID: 25702838

[10] Hanna S Etzioni A. MHC class I and II deficiencies. J Allergy Clin Immunol. (2014) 134(2):269–75. doi: 10.1016/j.jaci.2014.06.001, PMID: 25001848

[11] Zimmer J Donato L Hanau D Cazenave JP Tongio MM Moretta A. Activity and phenotype of natural killer cells in peptide transporter (TAP)-deficient patients (type I bare lymphocyte syndrome). J Exp Med. (1998) 187(1):117–22. doi: 10.1084/jem.187.1.117, PMID: 9419217 PMC2199183

[12] van Hateren A Elliott T. The role of MHC I protein dynamics in tapasin and TAPBPR-assisted immunopeptidome editing. Curr Opin Immunol. (2021) 270:138–43. doi: 10.1016/j.coi.2021.06.016, PMID: 34265495

[13] Michel T Poli A Zimmer J. Human leukocyte antigen class I deficiencies. Clin Immunol. (2017) 179:64–5. doi: 10.1016/j.clim.2017.03.009, PMID: 28344098

[14] Sleiman M Brons NH Kaoma T Dogu F Villa-Forte A Lenoble P. NK cell killer Ig-like receptor repertoire acquisition and maturation are strongly modulated by HLA class I molecules. J Immunol. (2014) 192(6):2602–10. doi: 10.4049/jimmunol.1302843, PMID: 24554773

[15] Gao Y Arkwright PD Carter R Cazaly A Harrison RJ Mant A. Bone marrow transplantation for MHC class I deficiency corrects T-cell immunity but dissociates natural killer cell repertoire formation from function. J Allergy Clin Immunol. (2016) 138(6):1733–6 e2. doi: 10.1016/j.jaci.2016.06.029, PMID: 27614800 PMC5138155

[16] Darazam IA Hakamifard A Momenilandi M Materna M Gharehbagh FJ Shahrooei M. Delayed diagnosis of chronic necrotizing granulomatous skin lesions due to TAP2 deficiency. J Clin Immunol. (2023) 43(1):217–28. doi: 10.1007/s10875-022-01374-7, PMID: 36227411

[17] Villa-Forte A de la Salle H Fricker D Hentges F Zimmer J. HLA class I deficiency syndrome mimicking Wegener's granulomatosis. Arthritis Rheumatol. (2008) 58(8):2579–82. doi: 10.1002/art.23675, PMID: 18668571

[18] Caversaccio M Bonel HM Carter R Williams AP Gadola SD. TAP deficiency syndrome: chronic rhinosinusitis and conductive hearing loss. Eur Arch Otorhinolaryngol. (2008) 265(10):1289–92. doi: 10.1007/s00405-008-0610-3, PMID: 18283480

[19] Zimmer J Poli A Andres E Hanau D Brons NH Hentges F. Reduced cytokine-mediated up-regulation of HLA-DR in TAP-deficient fibroblasts. Immunol Lett. (2006) 107(2):109–18. doi: 10.1016/j.imlet.2006.07.010, PMID: 16956670

[20] Bodemer C Sauvage V Mahlaoui N Cheval J Couderc T Leclerc-Mercier S. Live rubella virus vaccine long-term persistence as an antigenic trigger of cutaneous granulomas in patients with primary immunodeficiency. Clin Microbiol Infect. (2014) 20(10):O656–63. doi: 10.1111/1469-0691.12573, PMID: 24476349

[21] Perelygina L Plotkin S Russo P Hautala T Bonilla F Ochs HD. Rubella persistence in epidermal keratinocytes and granuloma M2 macrophages in patients with primary immunodeficiencies. J Allergy Clin Immunol. (2016) 138(5):1436–9 e11. doi: 10.1016/j.jaci.2016.06.030, PMID: 27613149 PMC5392721

[22] Neven B Perot P Bruneau J Pasquet M Ramirez M Diana JS. Cutaneous and visceral chronic granulomatous disease triggered by a rubella virus vaccine strain in children with primary immunodeficiencies. Clin Infect Dis. (2017) 64(1):83–6. doi: 10.1093/cid/ciw675, PMID: 27810866

[23] Buchbinder D Hauck F Albert MH Rack A Bakhtiar S Shcherbina A. Rubella virus-associated cutaneous granulomatous disease: a unique complication in immune-deficient patients, not limited to DNA repair disorders. J Clin Immunol. (2019) 39(1):81–9. doi: 10.1007/s10875-018-0581-0, PMID: 30607663 PMC7739844

[24] Wang Q Su H Han J Yang J Lin N. Case report: Rubella virus-associated cutaneous granuloma in an adult with TAP1 deficiency. Front Immunol. (2024) 15:1366840. doi: 10.3389/fimmu.2024.1366840, PMID: 38680488 PMC11045939

[25] Tezcan İ BA Ersoy F Sanal Ö. Sağlıklı Türk çocukları ve erişkinlerde türbimetrik yöntemle bakılan serum immünglobülin düzeyleri. Çocuk Sağlığı ve Hastalıkları Dergisi. (1996) 39:649–56.

[26] Ikinciogullari A Kendirli T Dogu F Egin Y Reisli I Cin S. Peripheral blood lymphocyte subsets in healthy Turkish children. Turk J Pediatr. (2004) 46(2):125–30., PMID: 15214740

[27] Dogu F Ikinciogullari A Fricker D Bozdogan G Aytekin C Ileri M. A novel mutation for TAP deficiency and its possible association with Toxoplasmosis. Parasitol Int. (2006) 55(3):219–22. doi: 10.1016/j.parint.2006.02.003, PMID: 16624613

[28] Pamer E Cresswell P. Mechanisms of MHC class I–restricted antigen processing. Annu Rev Immunol. (1998) 16:323–58. doi: 10.1146/annurev.immunol.16.1.323, PMID: 9597133

[29] Zhao J Li R Li Y Chen J Feng F Sun C. Broadly antiviral activities of TAP1 through activating the TBK1-IRF3-mediated type I interferon production. Int J Mol Sci. (2021) 22(9):4668. doi: 10.3390/ijms22094668, PMID: 33925089 PMC8125511

[30] Ramalingam TR Vaidhyanathan L Nk HR Uppuluri R Raj R. Clinical, immunological, and molecular findings in two patients with MHC class I deficiency and post-transplant outcome. Pediatr Allergy Immunol. (2024) 35(7):e14196. doi: 10.1111/pai.14196, PMID: 38989814

[31] de la Salle H Saulquin X Mansour I Klayme S Fricker D Zimmer J. Asymptomatic deficiency in the peptide transporter associated to antigen processing (TAP). Clin Exp Immunol. (2002) 128(3):525–31. doi: 10.1046/j.1365-2249.2002.01862.x, PMID: 12067308 PMC1906261

[32] Shields BE Perelygina L Samimi S Haun P Leung T Abernathy E. Granulomatous dermatitis associated with rubella virus infection in an adult with immunodeficiency. JAMA Dermatol. (2021) 157(7):842–7. doi: 10.1001/jamadermatol.2021.1577, PMID: 34037685 PMC8156178

[33] Wanat KA Perelygina L Chen MH Hao L Abernathy E Bender NR. Association of persistent rubella virus with idiopathic skin granulomas in clinically immunocompetent adults. JAMA Dermatol. (2022) 158(6):626–33., PMID: 35338705 10.1001/jamadermatol.2022.0828 PMC8957700

[34] Perelygina L Faisthalab R Abernathy E Chen MH Hao L Bercovitch L. Rubella virus infected macrophages and neutrophils define patterns of granulomatous inflammation in inborn and acquired errors of immunity. Front Immunol. (2021) 12:796065. doi: 10.3389/fimmu.2021.796065, PMID: 35003119 PMC8728873

[35] Luciani L Darazam IA Nougairède A Robert S Momenilandi M Rosain J. Serological diagnosis of chronic skin granulomas caused by wild-type or vaccine-derived rubella virus in patients with inherited HLA class I deficiency. J Hum Immun. (2025) 1(3):e20250103. doi: 10.70962/jhi.20250103 PMC12795598 41531537

[36] Perelygina L M-h C Suppiah S Adebayo A Abernathy E Dorsey M. Infectious vaccine-derived rubella viruses emerge, persist, and evolve in cutaneous granulomas of children with primary immunodeficiencies. PloS Pathog. (2019) 15(10):e1008080. doi: 10.1371/journal.ppat.1008080, PMID: 31658304 PMC6837625

[37] Zhang D Wanat KA Perelygina L Rosenbach M Haun PL Drolet BA. Cutaneous granulomas associated with rubella virus: A clinical review. J Am Acad Dermatol. (2024) 90(1):111–21. doi: 10.1016/j.jaad.2023.05.058, PMID: 37271455 PMC11887995

[38] Sherman FE Michaels RH Kenny FM. Acute encephalopathy (encephalitis) complicating rubella. report of cases with virologic studies, cortisol-production determinations, and observations at autopsy. JAMA. (1965) 192:675–81. doi: 10.1001/archpedi.1965.02090030390005, PMID: 14280514

[39] Tingle AJ Allen M Petty RE Kettyls GD Chantler JK. Rubella-associated arthritis. I. Comparative study of joint manifestations associated with natural rubella infection and RA 27/3 rubella immunisation. Ann Rheum Dis. (1986) 45(2):110–4. doi: 10.1136/ard.45.2.110, PMID: 3947141 PMC1001829

[40] Abernathy E Peairs RR Chen MH Icenogle J Namdari H. Genomic characterization of a persistent rubella virus from a case of Fuch' uveitis syndrome in a 73 year old man. J Clin Virol. (2015) 69:104–9. doi: 10.1016/j.jcv.2015.06.084, PMID: 26209390 PMC5873582

[41] Kreps EO Derveaux T De Keyser F Kestelyn P. Fuchs' uveitis syndrome: no longer a syndrome? Ocul Immunol Inflamm. (2016) 24(3):348–57. doi: 10.3109/09273948.2015.1005239, PMID: 26222767

[42] Tsilifis C MD Marques L Neves E Slatter MA Gennery AR. Stem cell transplantation as treatment for major histocompatibility class I deficiency. Clin Immunol. (2021) 229:108801. doi: 10.1016/j.clim.2021.108801, PMID: 34280577

[43] Maeda H Hirata R Chen RF Suzaki H Kudoh S Tohyama H. Defective expression of HLA class I antigens: a case of the bare lymphocyte without immunodeficiency. Immunogenetics. (1985) 21(6):549–58. doi: 10.1007/BF00395879, PMID: 3891604

[44] Sakuragi S Liao H Yajima K Fujiwara S Nakamura H. Rubella virus triggers type I interferon antiviral response in cultured human neural cells: involvement in the control of viral gene expression and infectious progeny production. Int J Mol Sci. (2022) 23(17):9799. doi: 10.3390/ijms23179799, PMID: 36077193 PMC9456041

[45] Chen TT Tsai MH Kung JT Lin KI Decker T Lee CK. STAT1 regulates marginal zone B cell differentiation in response to inflammation and infection with blood-borne bacteria. J Exp Med. (2016) 213(13):3025–39. doi: 10.1084/jem.20151620, PMID: 27849553 PMC5154933

[46] Nemoto K Kawanami T Hoshina T Ishimura M Yamasaki K Okada S. Impaired B-cell differentiation in a patient with STAT1 gain-of-function mutation. Front Immunol. (2020) 11:557521. doi: 10.3389/fimmu.2020.557521, PMID: 33133069 PMC7550620

[47] Romberg N Morbach H Lawrence MG Kim S Kang I Holland SM. Gain-of-function STAT1 mutations are associated with PD-L1 overexpression and a defect in B-cell survival. J Allergy Clin Immunol. (2013) 131(6):1691–3. doi: 10.1016/j.jaci.2013.01.004. Erratum in: J Allergy Clin Immunol. 2013 Dec;132(6):1460., PMID: 23403048 PMC3672340

[48] Kobbe R Kolster M Fuchs S Schulze-Sturm U Jenderny J Kochhan L. Common variable immunodeficiency, impaired neurological development and reduced numbers of T regulatory cells in a 10-year-old boy with a STAT1 gain-of-function mutation. Gene. (2016) 586(2):234–8. doi: 10.1016/j.gene.2016.04.006, PMID: 27063510

[49] Perelygina L Buchbinder D Dorsey MJ Eloit M Hauck F Hautala T. Outcomes for nitazoxanide treatment in a case series of patients with primary immunodeficiencies and rubella virus-associated granuloma. J Clin Immunol. (2019) 39(1):112–7. doi: 10.1007/s10875-019-0589-0, PMID: 30680653 PMC6383808

[50] Perelygina L Icenogle J Sullivan KE. Rubella virus-associated chronic inflammation in primary immunodeficiency diseases. Curr Opin Allergy Clin Immunol. (2020) 20(6):574–81. doi: 10.1097/ACI.0000000000000694, PMID: 33044342 PMC7730704

[51] Faisthalab R Suppiah S Dorsey M Sullivan KE Icenogle J Perelygina L. Drug sensitivity of vaccine-derived rubella viruses and quasispecies evolution in granulomatous lesions of two ataxia-telangiectasia patients treated with Nitazoxanide. Pathogens. (2022) 11(3):33., PMID: 35335662 10.3390/pathogens11030338 PMC8955873

[52] Leclerc-Mercier S Moshous D Neven B Mahlaoui N Martin L Pellier I. Cutaneous granulomas with primary immunodeficiency in children: a report of 17 new patients and a review of the literature. J Eur Acad Dermatol Venereol. (2019) 33(7):1412–20. doi: 10.1111/jdv.15568, PMID: 30869812

[53] Samarkandy S Khafaji R Alshareef A. Type I bare lymphocyte syndrome with novel TAP1 and TAP2 pathogenic variants. JAAD Case Rep. (2024) 51:22–5. doi: 10.1016/j.jdcr.2024.05.042, PMID: 39345282 PMC11437240

[54] Gao Y Arkwright PD Carter R Cazaly A Harrison RJ Mant A. Bone marrow transplantation for MHC class I deficiency corrects T-cell immunity but dissociates natural killer cell repertoire formation from function. J Allergy Clin Immunol. (2016) 138(6):1733–1736.e2. doi: 10.1016/j.jaci.2016.06.029, PMID: 27614800 PMC5138155

