# Supplementary material for: Clinical and immunological spectrum of MHC class I deficiency: insights from a long-term cohort with two novel mutations
Source: Front Immunol. 2025 Oct 7;16:1675097. doi: 10.3389/fimmu.2025.1675097 (PMC12537883; doi:10.3389/fimmu.2025.1675097)
Supplement: Supplementary file 1 [file Table1.docx]

**Supplementary Figures 1**

**

**

**Supplementary Figure 1: Pedigrees and genetic analysis of the patients**

A complementation assay performed on EBV-transformed B cells from the first four patients (P1–P4) revealed a defect in TAP subunit 1 in P1 and P2, and a defect in the TAP2 gene in P3 and P4. For the remaining patients, genomic DNA was extracted from EDTA-anticoagulated blood samples, and all exons of the TAP1 and TAP2 genes were sequenced using the Illumina MiSeq platform. Data were analyzed with MiSeq Reporter and IGV (v2.15.2) software. Segregation analysis confirmed a homozygous TAP1 mutation in affected individuals, consistent with autosomal recessive inheritance.

**(A, B, C)** This variant was first identified in P1 and P2 [27] and subsequently detected in P5, P6, and P7 from Family 3, and in P10 from Family 5. It involves a cytosine-to-thymine substitution at nucleotide position 1312 (c.1312C>T) in exon 5 of the TAP1 gene, resulting in a nonsense mutation (p.Arg438). This mutation is predicted to lead to nonsense-mediated mRNA decay (NMD) or the production of a truncated, nonfunctional protein. Notably, the same variant has also been reported in a biallelic state in an unrelated family (PMID: 16624613).

**(D)** A homozygous cytosine insertion (c.1022dupC, chr6:32797806) in exon 7 of the TAP2 gene causes a frameshift at codon 340 (p.Leu341Profs11), located between transmembrane domains 7 and 8. This mutation was identified in P3 and P4. Segregation analysis showed that both parents were heterozygous carriers.*

**(E)**  Genetic findings in P7 and P8. A novel homozygous single nucleotide deletion (c.1569del, p.Gly525Aspfs*36) was identified in exon 9 of the TAP2 gene (chr6:32798109 CT>C). This frameshift mutation introduces a premature termination codon (PTC), which is predicted to trigger nonsense-mediated mRNA decay (NMD) or lead to the synthesis of a truncated, likely non-functional protein.

**(F)** A homozygous novel mutation was detected in the TAP1 gene c.781del p.(Gln261ArgfsTer28).
