# Supplementary material for: Clinical and immunological spectrum of MHC class I deficiency: insights from a long-term cohort with two novel mutations
Source: Front Immunol. 2025 Oct 7;16:1675097. doi: 10.3389/fimmu.2025.1675097 (PMC12537883; doi:10.3389/fimmu.2025.1675097)
Supplement: Supplementary file 2 [file Table2.docx]

**SUPPLEMENTARY FIGURES (2-14)**


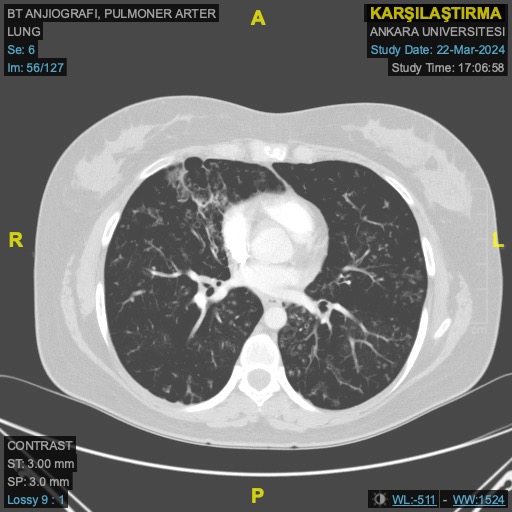


**Fig. S2:** Chest CT of P7 demonstrates diffuse bronchiectasis, mucus plugging, tree-in-bud nodules, and right middle lobe volume loss with atelectasis


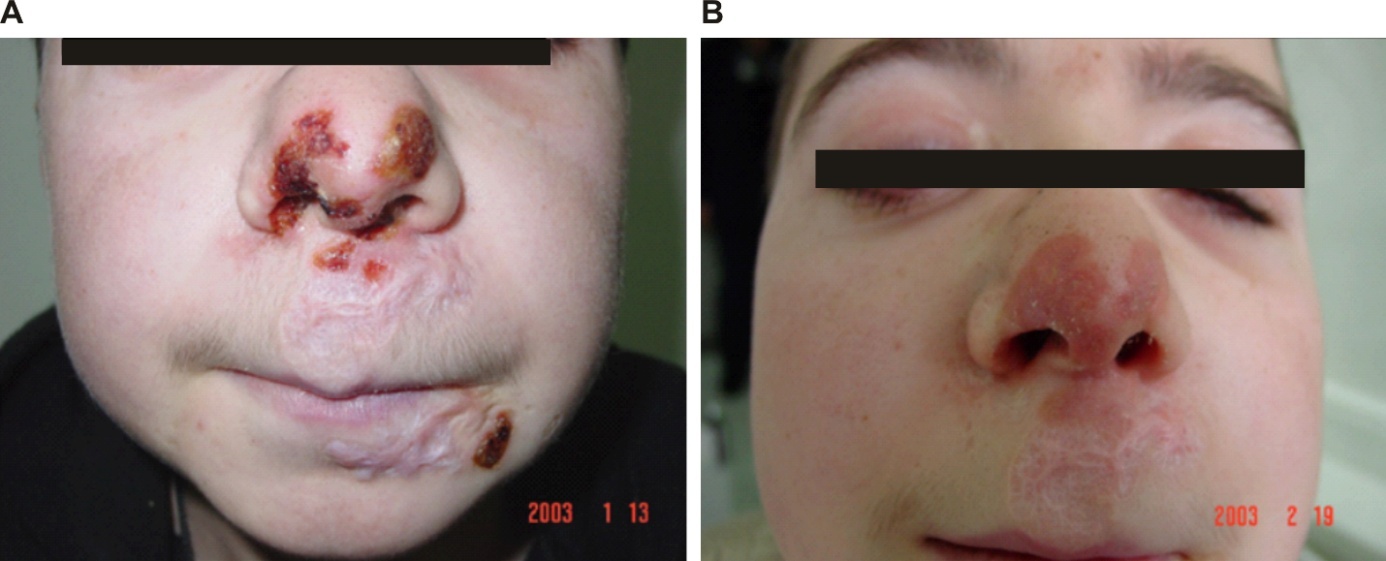


**Fig. S3.** Granulomatous lesions and scars on the mouth and nose of P1


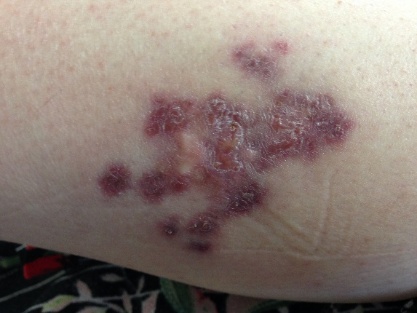

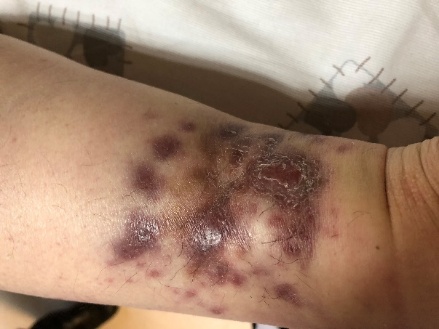

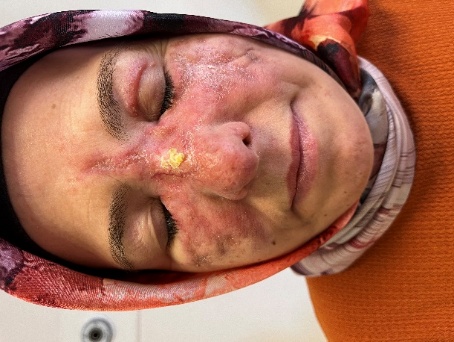


**Fig. S4.** Progression of cutaneous granulomatous lesions in patient P2. **a)** Ulcerative skin lesions on the leg first noted at the age of 32. **b)** At the age of 36, new skin lesions developed on the lower leg. **c)** Severe granulomatous ulceration of the leg and extensive facial skin lesions involving the nasal and malar regions at age 41.


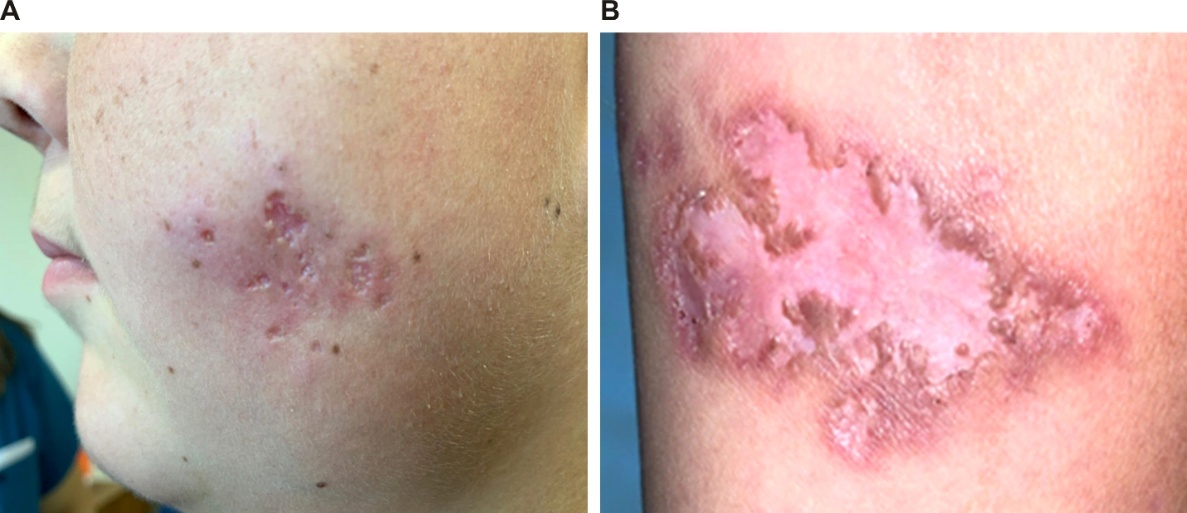


**Fig. S5.** Sharply marginated, pink-purple ulcerative skin lesions on the face and limbs observed in P10.


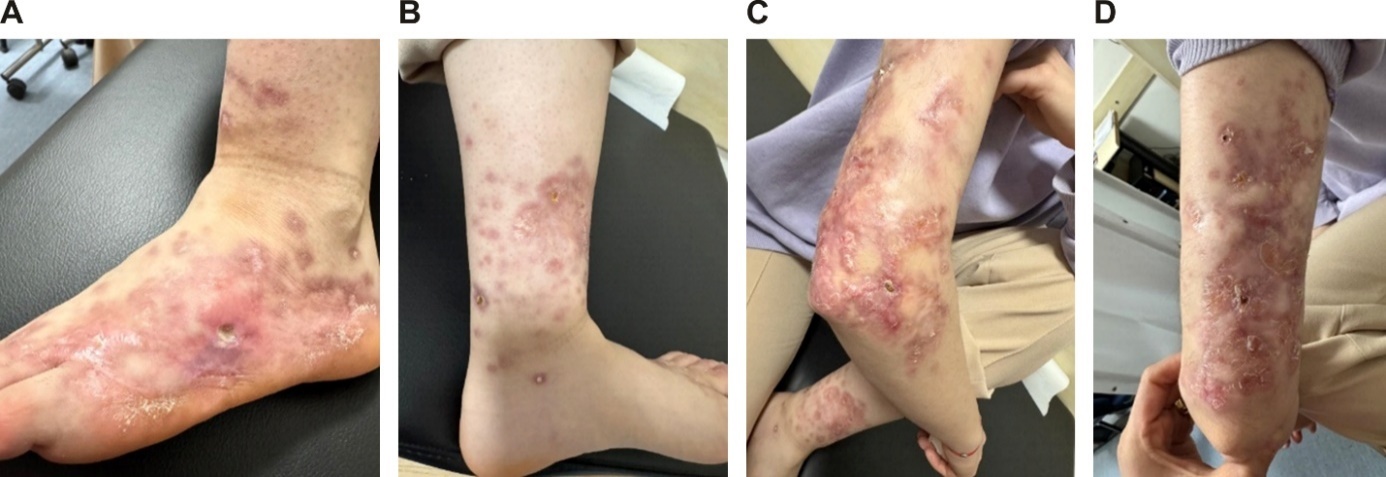


**Fig. S6.** Skin lesions on the dorsal surface of the right foot, the left leg, and both arms of P11.


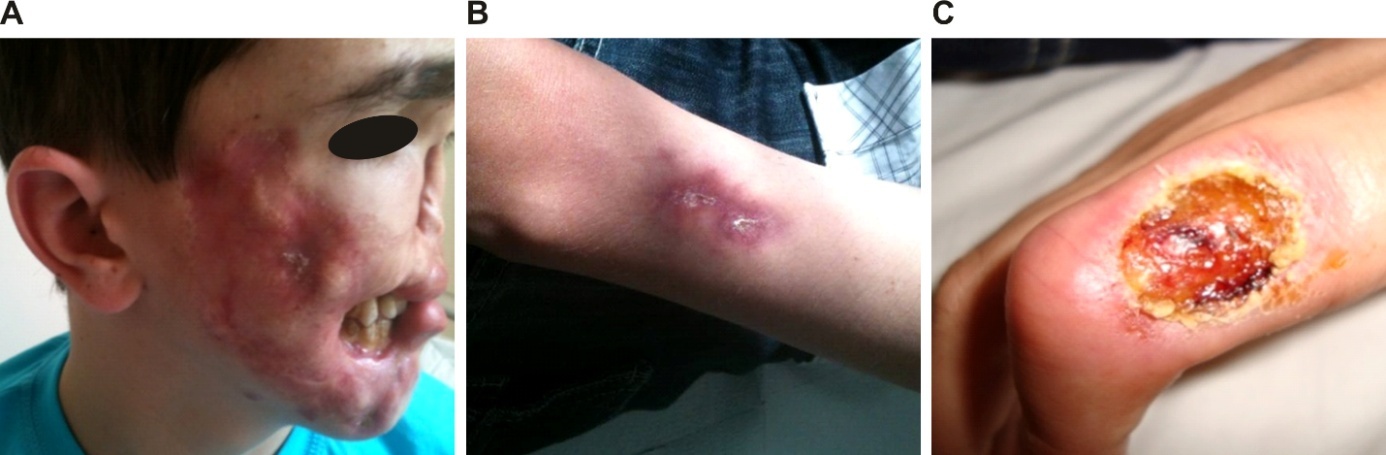


**Fig. S7.** Prior to mesenchymal stem cell (MSC) therapy, P3 had lesions on the face, nose, lips, arm, and fingers.


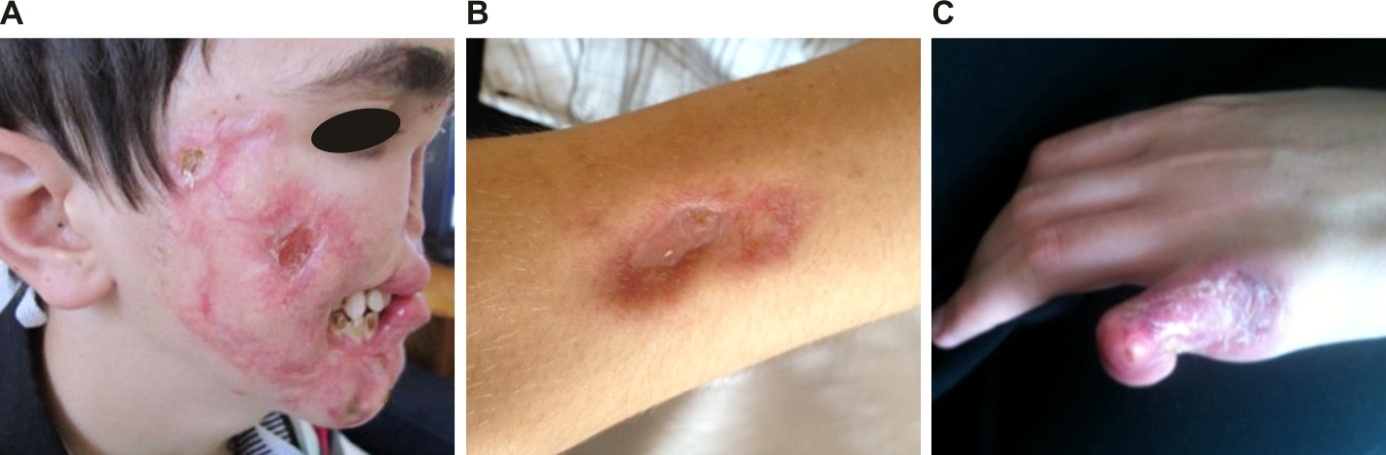
**Fig. S8.** After MSC therapy, partial improvement was observed in the lesions on the P3's face, nose, lips, arm, and fingers.


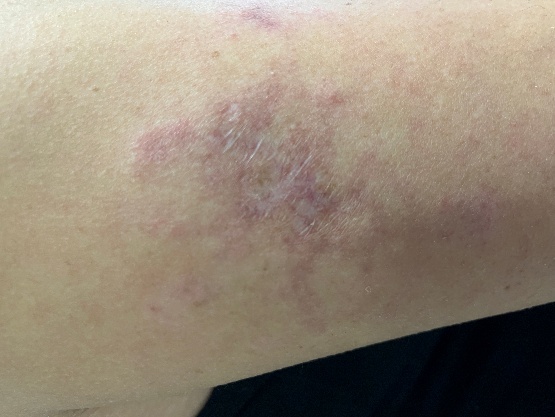

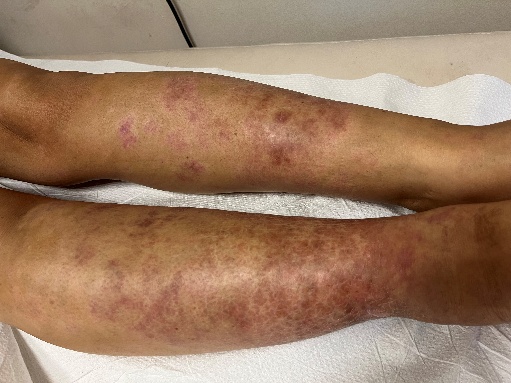


**Fig. S9.** Granulomatous skin ulcers and vasculitic changes in P8. In addition to ulcerative lesions, vasculitic skin discoloration and edema were observed on the legs.


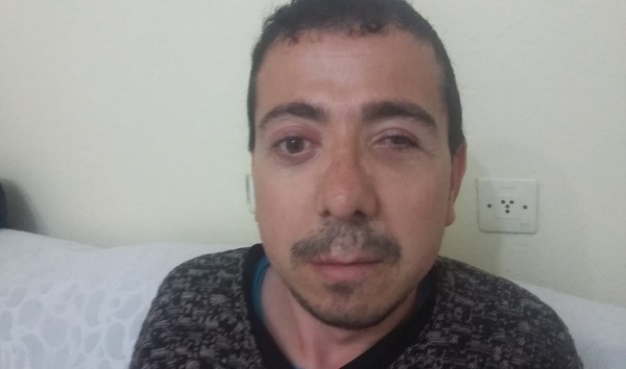


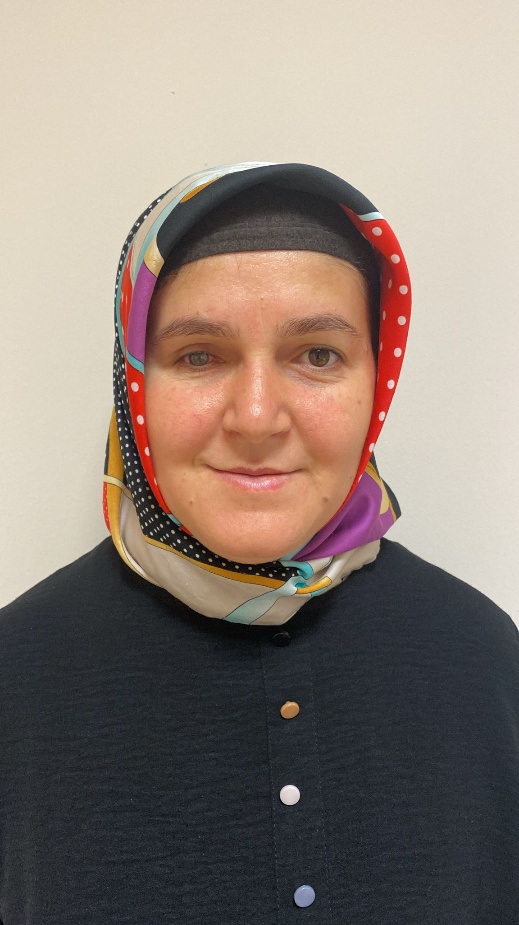


**Fig. S10.** Enophthalmos and heterochromia were observed in P1 and P4 as secondary findings of chronic uveitis


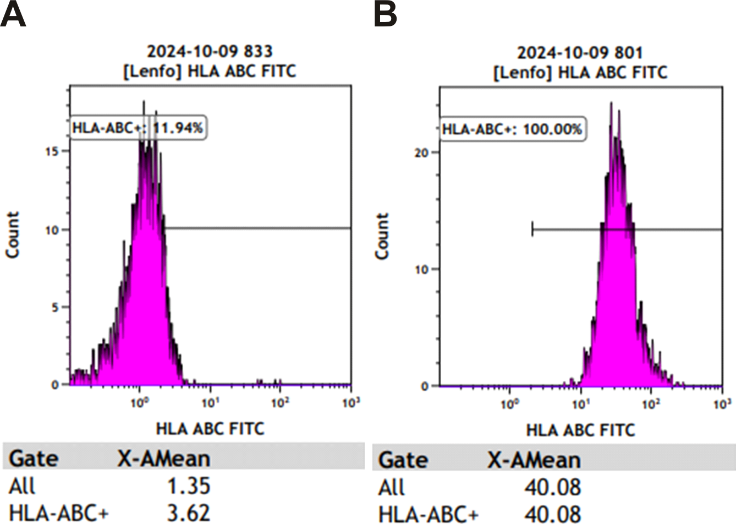


**Fig. S11.** HLA-ABC expression (11,9%) and MFI measurement (3,6) of P4. **(B)** HLA-ABC expression (100%) and MFI measurement (40,08) in healthy, age-matched control.

**A**

**B**

**Fig. S12. Type I IFN responses of P5 and age-matched control.**

**A)** Relative mRNA expression of IFN-α, IFN-β1, IFI44L, and IFIT1 in unstimulated (Unstim) and Poly I:C–stimulated PBMCs. Expression values are normalized to housekeeping genes. Each dot indicates a biological replicate; bars show mean ± SEM. *p < 0.05, **p < 0.01, ***p < 0.001, ****p < 0.0001.

**B)** qPCR analysis of RSAD2, IFI27, and CXCL9 in unstimulated (Unstim) and Poly I:C–stimulated (+) PBMCs from the patient (PT) and a healthy control (CTRL). Relative expression was normalized to housekeeping controls. Both patient and control represent single biological replicates, each analyzed in technical duplicate. Statistical analysis was performed using unpaired t-test

**A**

**B**

**Fig. S13. Type I IFN responses of P7 and age-matched control.**

**A)** Relative expression of IFN-α, IFN-β1, IFI44L, and IFIT1 under basal and Poly I:C–stimulated conditions, normalized to housekeeping genes.

**B)** qPCR of RSAD2, IFI27, and CXCL9 in unstimulated (−) and Poly I:C–stimulated (+) PBMCs. Data shown as single biological replicates with technical duplicates.

**A**

**B**

**Fig. S14. Type I IFN responses of P8 with granulomatous lesions.**

**A)** Elevated baseline expression of IFN-α, IFN-β1, IFI44L, RSAD2, IFI27, IL1β, and ISG15 was observed, with further induction after Poly I:C stimulation. Data represent single biological replicates analyzed in duplicate.

**B)** qPCR of TNFα, IL1β, NFκB, and SIGLEC1 in unstimulated (−) and Poly I:C–stimulated (+) PBMCs. Expression was normalized to housekeeping genes and shown on a log scale. Both PT and CTRL were single biological replicates analyzed in technical duplicate.
