# Supplementary material for: Clinical and immunological spectrum of MHC class I deficiency: insights from a long-term cohort with two novel mutations
Source: Front Immunol. 2025 Oct 7;16:1675097. doi: 10.3389/fimmu.2025.1675097 (PMC12537883; doi:10.3389/fimmu.2025.1675097)
Supplement: Supplementary file 3 [file Table3.docx]

**Supplementary Materials and Methods**

**Case Reports**

**P1-FI:** The patient, who is now 37 years old, was initially evaluated at the age of 14 for recurrent respiratory tract infections (RTIs), bronchiectasis, granulomatous skin lesions, and visual loss. He was the third child of a consanguineous family. At the age of 13, he developed midline facial ulcers, submandibular lymphadenopathy, and uveitis requiring ocular surgery. Physical exam showed facial scarring **(Figure S3)** and enophthalmos **(Figure S10)**. During hospitalization, he developed severe interstitial pneumonia. Extensive infectious workup including PCR for CMV, EBV, RSV, adenovirus, P. Jirovecii, M. Tuberculosis, and HIV serology was negative. Chest CT showed diffuse interstitial infiltrates. Following the detection of positive anti-Toxoplasma IgM and IgG, the patient’s retinitis and pneumonia were attributed to toxoplasmosis, and pyrimethamine–sulphadoxin and folinic acid were initiated, resulting in a decline in IgM titers. Flow cytometry showed markedly reduced HLA-ABC expression. Genetic analysis revealed a homozygous C>T mutation in the TAP1 gene, resulting in a premature stop codon, as previously reported by Doğu et al. (27). Immunoglobulin replacement therapy (IgRT) and trimethoprim-sulfamethoxazole (TMP-SMX) prophylaxis were started. Despite recurrent pneumonias, infections were controlled with IV antibiotics. Chest CT showed diffuse bronchiectasis, and PFTs revealed severe restriction. Skin lesions did not recur. P. Aeruginosa colonization was persistent over the past 5 years, managed with dual antipseudomonal antibiotics during exacerbations and monthly inhaled tobramycin. At the time of writing this article, the patient had experienced a sudden loss of vision in his right eye. Uveitis and glaucoma were diagnosed. The aqueous humour sample (PCR) test revealed a negative result for HSV1, HSV2, CMV, EBV, VZV, Adenovirus, Toxoplasma, and Rubella virus. Screening for tuberculosis and sarcoidosis (PPD, Quantiferon, ACE level) yielded negative results. A response was observed in the patient following high-dose steroid treatment.

**P2-FI:** A 41-year-old woman was evaluated at the age of 21 after her brother (P1) was diagnosed with MHC class I deficiency. HLA-ABC expression was measured at 2 %. Since the age of 10, she had recurrent RTIs. Chest CT revealed bilateral diffuse bronchiectasis. She received IgRT and TMP-SMX prophylaxis for 10 years with clinical stability. At the age of 32, two scarring skin lesions appeared on the back of her left leg and right ankle, measuring 8x10 cm and 6x8 cm, respectively. These lesions were dark pink-purple in color. Skin ulcers were resistant to topical steroids and rivanol. New ulcerative lesions appeared on the face and hard palate, with biopsy showing granulation tissue and pustulating fistula; no pathogens were isolated. Ulceration extended to nasal dorsum, leading to anterior nasal septum perforation. **(Figure S4 a-c)** Swab cultures grew *methicillin-sensitive Staphylococcus aureus* (MSSA). Skin biopsy showed granulomatous inflammation without fungal or acid-fast bacilli in two different skin biopsies which were performed in the consecutive two years. The inflammation had features reminiscent of necrobiotic (collagenolitic) granulomas such as collagenolytic nature of the necrosis seen in some granulomas and vasculitic/vasculopathic changes in the neighborhood of necrosis. **(Figure 2 a-c)** The microscopic appearance especially has recalled necrobiosis lipoidica. Anti-rubella IgM remained positive for 5 years. PPD was positive, likely from prior BCG vaccination; BAL cultures and PCR for pathogens including *P. jirovecii* were negative. She was treated with systemic and local antibiotics. At age 36, pneumonia recurred with worsening bronchiectasis and *Aspergillus fumigatus* isolated from BAL; posaconazole was started. She had moderate obstructive and restrictive PFT changes. Inhaling steroids and LABA showed partial clinical improvement. She continues on IgRT, TMP-SMX, posaconazole, and topical skin therapies.

**P3-FII:** A 10-year-old boy, born to consanguineous parents, was referred at age 9 for ulcerative facial lesions. A skin biopsy suggested lupus vulgaris, but tuberculosis (TB) screening (PPD, ARB smear, imaging) was negative. A sister had died previously with similar lesions during presumed TB treatment. Based on history and biopsy, empirical anti-TB therapy was given for six months without improvement. Immunological evaluation showed low IgM, and reduced HLA-ABC expression (5%). Complementation assays confirmed a TAP2 defect, and Sanger sequencing revealed a homozygous TAP2 mutation, establishing the diagnosis of MHC class I deficiency. IgRT and TMP-SMX prophylaxis were initiated. Paranasal CT showed nasal cartilage and bone destruction with skin fistulization. Progressive ulcerations developed on the face, palate, cheek, and right foot. Cultures revealed *Streptococcus*, *S. Aureus*, and later *P. Aeruginosa*. Despite IV antibiotics, topical care, and surgical debridement, lesions worsened. By age 15, nasal destruction and hard palate perforation had occurred **(Figure 1 a-c).** Hearing loss developed due to Eustachian tube obstruction and otomastoiditis; surgical interventions included ventilation tube and nasal and palatal prostheses. Ulcerations later spread and worsened despite further antibiotics, grafting, and two autologous and two allogeneic mesenchymal stem cell (MSC) infusions, which offered only transient benefit **(Figure S7 and S8).** Candida esophagitis developed and impaired feeding. At age 21, wound cultures grew *P. Aeruginosa*, *A. Baumannii*, *S. Aureus*, and *C. glabrata*, and targeted antimicrobial treatments were given. He developed autoamputation of the fifth finger. HSCT was considered, but no matched donor was found. He eventually died due to sepsis and multiorgan failure.

**P4-FII.** A 34-year-old female was first evaluated at age 15 during family screening following her brother’s (P3) diagnosis of MHC class I deficiency. Aside from 2–3 episodes of RTIs per year and a history of moderate varicella at age 7, she had no major complaints. However, chest CT revealed bronchiectasis and pneumonic infiltrates. Sputum culture grew *S. Pneumoniae*, and she was treated with IV antibiotics. Vaccine responses and isohemagglutinin titers were normal. HLA-ABC expression was 7%. TMP-SMX prophylaxis were initiated. At age 21, she was hospitalized for otitis, sinusitis, and pneumonia. She developed right eye pain and photophobia. Ophthalmologic examination revealed a granulomatous lesion that was thought to be consistent with herpes virus infection; acyclovir was initiated. Serologies for toxoplasma, hepatitis, syphilis, and EBV were negative, however rubella IgM and IgG were positive. Chronic uveitis, and glaucoma developed in the right eye. She was treated with topical steroids, valganciclovir, and dorzolamide. At age 26, she underwent cataract surgery and trabeculotomy. She was lost to follow-up for eight years and didn’t receive regular treatment. At 34, her last check-up revealed chronic respiratory symptoms, right eye vision loss, enophthalmos, and heterochromia **(Figure S10),** and abnormal lung and nasal findings.

**P5-FIII**: A 19-year-old male, third child of consanguineous parents, was first evaluated at age two for recurrent RTIs. Chest CT and bronchoscopy and revealed bronchiectasis and lower lobe infiltrates. Immunologic work-up showed low IgA and normal sweat and reflux tests. CMV viremia (7900 copies/mL) was treated with ganciclovir. He was lost to follow-up for ten years, then returned at 13 with chronic coughs, RTIs and hearing loss. HLA-ABC expression was 13%, CD8⁺ T cells were reduced (10%), and TCR γδ⁺ cells elevated (12%). His older brother (P6) also had low HLA-ABC expression and chronic respiratory symptoms. Next-generation sequencing (NGS) analysis revealed a homozygous TAP1 mutation in both siblings; their parents and sister were found to be heterozygous carriers. IgRT, TMP-SMX, inhaled steroids, and LABA were started. At age 18, he was hospitalized with cough, hemoptysis, and hypoxia. Thoracic CT showed bronchiectasis and mucus plugging. *Aspergillus fumigatus* was isolated from BAL and sputum. Amphotericin B and voriconazole was initiated, and prophylaxis continued. One year later, he was rehospitalized with pneumonia, but no pathogen was identified. Chest CT showed progression of bronchiectasis. At his last checkup, he had pneumonia and a dental abscess, treated with oral antibiotics.

**P6-FIII**. The older brother of P5 presented at age 16 with chronic cough and RTIs since early childhood. His history included multiple antibiotic courses, five tympanostomy tube placements, and nasal polyp surgery. Immunologic evaluation revealed low IgM and selective IgA deficiency. HLA-ABC expression was reduced (22%). IgRT and TMP-SMX prophylaxis were initiated. During the follow-up, he did not have any serious infection except recurrent RTIs and a dental abscess. Chest CT showed slowly progressing centriacinar nodules over six years, without bronchiectasis or skin involvement.

**P7-FIII.** A 27-year-old woman, cousin of P5 and P6, was born to consanguineous parents. Since infancy, she had recurrent RTIs, pneumonia, chronic bronchitis and asthma attacks requiring multiple hospitalizations. At age 15, she was diagnosed with bronchiectasis and started on inhaled steroids. At her initial evaluation at age 19, she exhibited open-mouth breathing, rhinolalia, purulent nasal discharge, small tonsils, diffuse rales, and diminished breath sounds. Sputum culture grew *Actinobacillus ureae*, treated with levofloxacin. Chest CT showed diffuse bronchiectasis, mucus plugging, tree-in-bud nodules, and right middle lobe volume loss with atelectasis **(Figure S2).** PFTs were severely impaired (FVC: 31%, FEV1: 20%) but remained stable during follow-up. Immunologic workup showed normal immunoglobulins, negative anti-HBs and isohemagglutinins, with 10% HLA-ABC expression. Genetic testing confirmed a homozygous TAP1 c.1312C>T (p.R438*) mutation, also present in her cousins (P5 and P6). IgRT and TMP-SMX prophylaxis were initiated. No HLA-matched family donor was identified for HSCT.

**P8-FIV.** A 41-year-old woman born to consanguineous parents, was referred at age 33 for evaluation of an IEI. She had a history of RTIs since early childhood and had been misdiagnosed with asthma and Familial Mediterranean Fever (FMF), receiving inhalers and colchicine. At age six, she developed hydrocephalus presumed to be post-measles, requiring ventriculoperitoneal shunt placement. At 25, she developed chronic right eye uveitis, treated with hydroxychloroquine and adalimumab; recurrent attacks led to surgery. At first evaluation, she had small tonsils, heterochromia with mild microphthalmia of the right eye**,** and multiple ulcerated skin lesions. Immunologic tests revealed normal immunoglobulins and lymphocyte counts, but severely reduced HLA-ABC expression (6%, MFI 2.3). Genetic analysis identified a homozygous *TAP2* mutation. She was started on IgRT and TMP-SMX prophylaxis. Skin biopsy revealed granulomatous inflammation with CD8+ T-cell predominance and B-cell depletion; stains and serologies for infectious agents were negative Like P2 patient focal vasculopathic changes near the necrobiosis area were present. In addition mild superficial subcutaneous adipose tissue inflammation was seen**.** During 7 years of follow-up, she had chronic sinusitis without severe infections. At her last visit, nasal bone perforation and violaceous ulcerated leg lesions due to venous insufficiency were observed, and treatment with calcium dobesilate and aspirin was initiated. Anti-Rubella IgM was initially negative but later became positive (10.6 COI, negative:<1 COI). She remains on hydroxychloroquine and topical steroids for uveitis.

**P9- FIV.** A 44-year-old male, the older brother of P8, had recurrent RTIs and chronic ear discharge since age 10, with measles in childhood concurrent with his sister. In adulthood, he experienced persistent ear discharge, postnasal drip, and needed frequent antibiotics. He also had occasional oral candidiasis. Thoracic CT showed upper lobe nodular infiltrates and left lower lobe bronchiectasis. Immunologic workup revealed low HLA-ABC expression (42%, MFI 3.7), and genetic testing confirmed the same TAP2 mutation as P8. HLA typing showed a shared haplotype. Serological tests were performed before the start of IgRT. Anti-measles IgG was very high at 2,202 mIU/mL (a positive value is >250 mIU/mL in the healthy population), while anti-measles IgM was negative. Anti-rubella IgG was >10,000 COI (a positive value is >10 COI in the healthy population) and anti-rubella IgM was 7.79 COI (a positive value is >1 COI).

**P10-FV.** A 19-year-old male was first evaluated for suspected IEI at age 13 due to recurrent bronchiolitis and pneumonia since age two, previously diagnosed as asthma. At age 12, ulcerated skin lesions appeared on his face and extremities **(Figure S5)**. Examination revealed coarse rales and multiple ulcerative skin lesions. Immunologic tests showed mildly low IgM, negative anti-HBs, reduced CD3⁺, CD8⁺, CD19⁺, and SMB cells, and absent HLA-ABC expression (0%). A homozygous TAP1 mutation was identified. PFTs showed a restrictive pattern. He is hospitalized 3–4 times per year for pneumonia. Thoracic CT revealed diffuse bronchiectasis, tree-in-bud pattern, and acinar infiltrates. BAL cytology showed acute suppurative inflammation; sputum culture grew *Rothia mucilaginosa*. Skin biopsy showed granulomatous dermatitis with caseous necrosis, without detectable pathogens. Anti-Rubella IgM was positive, but serum rubella PCR was negative. He remains under follow-up for ongoing respiratory and skin involvement.

**P11-FVI**. A 23-year-old woman developed skin lesions on her arms and ankles at age 11 **(Figure S6).** Initial biopsy was inconclusive; she received empirical antibiotics and corticosteroids. A second biopsy at 14 showed granulomatous dermatitis, leading to anti-tuberculosis treatment and antibiotics for recurrent sinusitis and bronchitis. Due to lesion progression, she was referred to immunology. Her non-consanguineous parents originated from neighboring villages; she has two healthy siblings. Immunologic workup revealed mildly low IgM and CD19⁺ B cells, with HLA-ABC expression reduced to 5%. A homozygous TAP1 mutation was detected. Anti-rubella IgG and IgM were positive before IgRT. Family screening showed normal HLA class I expression in parents and siblings. She was diagnosed with MHC class I deficiency and started on IgRT and TMP-SMX prophylaxis.

**Functional Immunological Assays**

**T-cell Activation with PHA and anti-CD3**

Peripheral blood mononuclear cells (PBMCs) were isolated from heparinized blood samples using Ficoll-Paque™ density gradient centrifugation. After two washes with RPMI 1640, cells were resuspended at 2×10⁶ cells/mL in RPMI 1640 supplemented with 10% fetal calf serum (FCS). Cells were stimulated with phytohemagglutinin (PHA, 10 µg/mL) or anti-CD3 monoclonal antibody (10 µg/mL) in 24-well plates and incubated for 48 hours at 37 °C in 5% CO₂. Following incubation, cells were stained with fluorochrome-conjugated monoclonal antibodies against CD3, CD4, CD25, and CD69. Flow cytometric analysis was conducted using a Navios EX FlowCytometer (Beckman Coulter Corp. Miami FL, USA). A minimum of 10,000 gated lymphocyte events were collected. Data were analyzed using appropriate flow cytometry software.

**Neutrophil Oxidative Burst Assay (DHR 123 Test)**

Neutrophil respiratory burst activity was assessed using dihydrorhodamine 123 (DHR 123) and phorbol 12-myristate 13-acetate (PMA). Briefly, 100 µL of EDTA-anticoagulated whole blood was added to two tubes labeled as unstimulated (resting) and stimulated. PMA working solution (25 µL) was added to the stimulated tube, and PBS (25 µL) was added to the unstimulated tube. After gentle mixing, both tubes were incubated at 37 °C for 15 minutes. Then, 25 µL of DHR 123 working solution was added to each tube, followed by a second 15-minute incubation at 37 °C. Following incubation, red blood cells were lysed and the cells were fixed according to standard protocols. Flow cytometric analysis was performed on a Navios EX FlowCytometer (Beckman Coulter Corp. Miami FL, USA), and a minimum of 30,000 gated events were acquired per sample.

**Rubella Serology (IgG and IgM)**

Rubella virus-specific IgG and IgM antibodies were analyzed using the Elecsys Rubella IgG (catalog no: 07027770500) and Elecsys Rubella IgM (catalog no: 07027796500) assays (Roche Diagnostics GmbH, Mannheim, Germany), both performed on the cobas e 801 immunoassay analyzer (Roche Diagnostics). The assays are based on electrochemiluminescence immunoassay (ECLIA) technology.

For IgG detection, the assay utilizes a sandwich principle with recombinant E1 antigen, and for IgM, a μ-capture format is employed. Each assay includes streptavidin-coated microparticles, biotinylated anti-human IgG or IgM monoclonal antibodies, and ruthenylated anti-rubella antibodies.

All samples were processed according to the manufacturer's instructions. For IgM testing, specimens were automatically diluted 1:20 using Diluent Universal. Calibration was performed using RUBIGG Cal1/Cal2 (IgG) and RUBIGM Cal1/Cal2 (IgM), and internal quality control was carried out using PreciControl Rubella IgG and IgM controls.

Rubella IgG results were considered positive if ≥10 IU/mL, while IgM results were interpreted based on cut-off index (COI) values: COI ≥1.0 as positive, <0.8 as negative, and 0.8–1.0 as equivocal, requiring retesting if necessary.

**Type I IFN Assay**

Peripheral blood mononuclear cells (PBMCs) were isolated from heparinized peripheral blood samples by density gradient centrifugation using Ficoll-Paque™ PLUS (GE Healthcare, Cat# 17-1440-02). A total of 1 × 10⁶ PBMCs were seeded into 24-well plates and cultured in RPMI-1640 medium (Gibco) supplemented with 10% fetal bovine serum (FBS; Gibco) and 1% penicillin–streptomycin (Gibco). Cells were incubated for 24 hours at 37 °C in a humidified incubator with 5% CO₂ under two conditions: unstimulated and stimulated with 20 µg/mL Polyinosinic–polycytidylic acid [Poly I:C] (Sigma-Aldrich, Cat# P1530-100MG).

Total RNA was extracted using PureZOL™ RNA Isolation Reagent (Bio-Rad, Cat# 7326890) according to the manufacturer’s instructions. RNA purity and concentration were determined using a NanoDrop™ 2000 spectrophotometer (Thermo Fisher Scientific). For cDNA synthesis, 1 µg of total RNA was reverse-transcribed using the iScript™ cDNA Synthesis Kit (Bio-Rad, Cat# 1708891). Quantitative real-time PCR (qRT-PCR) was performed on a LightCycler® 480 System (Roche) using the SsoAdvanced™ Universal SYBR® Green Supermix (Bio-Rad, Cat# 1725274). 18S was used as the internal reference gene, and relative gene expression levels were calculated using the 2^–ΔΔCt method.

**Quantitative PCR Primer Sequences**

The following primer pairs were used to assess type I IFN signaling and interferon-stimulated gene (ISG) expression:

| **Gene** | **Forward Primer (5'-3')** | **Reverse Primer (5'-3')** |
| --- | --- | --- |
| TNFα | GAAAGCATGATCCGGGACGTG | GATGGCAGAGAGGAGGTTGAC |
| IFNα | CTGTGTGATGAGCTGACCAAAG | CTGCTGGTAGAGTTCGGTGC |
| IFNβ1 | GCTTGGATTCCTACAAAGAAGCAG | GTCTCATTCCAGCCAGTGCT |
| IF144L | TCTGTGGCAGGAAGAAGATGC | GTCTCCTGGAAGGATGTGGC |
| CXCL9 | GGCCTTGGAGCGAGGCAGTT | TTTTCAACCCCTCTTCCTGGGC |
| IFIT1 | GGTGGAAGATTGCTGAGGATGA | GGTTTCTGGCTCTGCTCTCTG |
| RSAD2 | AGCAGGTCAAGGAGGGAGAG | ATTCAGGGAGCGGAACTTCT |
| IFI27 | CTGCCAGTTTGAGGAAGCTG | TTTCCCTGAGGACCTGAGGT |
| IL6 | ACTCACCTCTTCAGAACGAATTG | CCATCTTTGGAAGGTTCAGGTTG |
| IL1β | ATGATGGCTTATTACAGTGGCAA | GTCGGAGATTCGTAGCTGGA |
| NFKβ1 | AGGAGGGACCTCACTGCTCA | CTTGTTGTTGGCATCAGGCT |
| SIGLEC1 | GTGGTTCCATGGTGTTGTTG | CCAGCTCCTGGATCTCCTTC |
| ISG15 | GAGAGGCAGCGAACTCATCT | CTTTATTTCCGGCCCTTGAT |
| 18s | GTAACCCGTTGAACCCCATT | CCATCCAATCGGTAGTAGCG |
